# Supplementary material for: Cost function dependent barren plateaus in shallow parametrized quantum circuits
Source: Nat Commun. 2021 Mar 19;12:1791. doi: 10.1038/s41467-021-21728-w (PMC7979934; doi:10.1038/s41467-021-21728-w)
Supplement: Supplementary file 1 — Supplementary Information [file 41467_2021_21728_MOESM1_ESM.pdf]

Supplementary Information for "Cost Function Dependent Barren Plateaus in Shallow Parametrized Quantum Circuits"

M. Cerezo,[1,2] Akira Sone,[1,2] Tyler Volkoff,[1] Lukasz Cincio,[1] and Patrick J. Coles[1]

[1] *Theoretical Division, Los Alamos National Laboratory, Los Alamos, NM, USA.*
[2] *Center for Nonlinear Studies, Los Alamos National Laboratory, Los Alamos, NM, USA*

In this Supplementary Information, we present detailed proofs of the propositions, theorems, and corollaries presented in the manuscript "Cost Function Dependent Barren Plateaus in Shallow Parametrized Quantum Circuits". In Supplementary Note 1 we first introduce several lemmas which will be useful in the derivation of our main results. Then, in Supplementary Note 2, and Supplementary Note 3 we respectively provide proofs for Propositions 1 and 2 of the main text. We then derive the general equations for the variance of the cost function partial derivative in Supplementary Note 4. In Supplementary Note 5 we explicitly evaluate the variance of the cost function derivative for the special case when $V(\boldsymbol{\theta})$ is given by a single layer of the Alternating Layered Ansatz.

In Supplementary Note 6, and Supplementary Note 7, we provide our proofs to Theorem 2 and Theorem 1, respectively. Where we remark that the proof of Theorem 2 comes before that of Theorem 1 since the latter builds on the former. Then, in Supplementary Note 8, we prove Corollaries 1, and 2. In Supplementary Note 9, we demonstrate that the local cost function for the quantum autoencoder is faithful.

Supplementary Note 1: Preliminaries

In this section, we present properties that allow for analytic calculation of integrals of polynomial functions over the unitary group with respect to the unique normalized Haar measure. For more details on this topic, we refer the reader to Ref. [1, 2]. In addition, to make the Supplementary Information more self-contained, we reiterate here the definition of a t-design. Consider a finite set $\{W_y\}_{y\in Y}$ (of size $|Y|$) of unitaries W_y on a d-dimensional Hilbert space, and let $P_{(t,t)}(W)$ be an arbitrary polynomial of degree at most t in the matrix elements of W and at most t in those of $W^\dagger$. Then, we say that this finite set is a t-deisgn if [3]

$$\frac{1}{|Y|}\cdot\sum_{y\in Y} P_{(t,t)}(W_y) = \int_{U(d)} d\mu(W) P_{(t,t)}(W)\,, \tag{1}$$

where in the right-hand side $U(d)$ denotes the unitary group of degree d. Equation (1) implies that averaging $P_{(t,t)}(W)$ over the t-design is indistinguishable from integrating over $U(d)$ with respect to the Haar distribution.

Given $W\in U(d)$ the following expressions are valid for the first two moments [1, 2]

$$\int_{U(d)} d\mu(W) w_{\boldsymbol{i},\boldsymbol{j}} w^*_{\boldsymbol{p},\boldsymbol{k}} = \frac{\delta_{\boldsymbol{i},\boldsymbol{p}}\delta_{\boldsymbol{j},\boldsymbol{k}}}{d}\,, \tag{2}$$

$$\begin{aligned}\int_{U(d)} d\mu(W) w_{\boldsymbol{i}_1,\boldsymbol{j}_1} w_{\boldsymbol{i}_2,\boldsymbol{j}_2} w^*_{\boldsymbol{i}'_1,\boldsymbol{j}'_1} w^*_{\boldsymbol{i}'_2,\boldsymbol{j}'_2} =& \frac{1}{d^2-1}\left(\delta_{\boldsymbol{i}_1,\boldsymbol{i}'_1}\delta_{\boldsymbol{i}_2,\boldsymbol{i}'_2}\delta_{\boldsymbol{j}_1,\boldsymbol{j}'_1}\delta_{\boldsymbol{j}_2,\boldsymbol{j}'_2}+\delta_{\boldsymbol{i}_1,\boldsymbol{i}'_2}\delta_{\boldsymbol{i}_2,\boldsymbol{i}'_1}\delta_{\boldsymbol{j}_1,\boldsymbol{j}'_2}\delta_{\boldsymbol{j}_2,\boldsymbol{j}'_1}\right)\\ &-\frac{1}{d(d^2-1)}\left(\delta_{\boldsymbol{i}_1,\boldsymbol{i}'_1}\delta_{\boldsymbol{i}_2,\boldsymbol{i}'_2}\delta_{\boldsymbol{j}_1,\boldsymbol{j}'_2}\delta_{\boldsymbol{j}_2,\boldsymbol{j}'_1}+\delta_{\boldsymbol{i}_1,\boldsymbol{i}'_2}\delta_{\boldsymbol{i}_2,\boldsymbol{i}'_1}\delta_{\boldsymbol{j}_1,\boldsymbol{j}'_1}\delta_{\boldsymbol{j}_2,\boldsymbol{j}'_2}\right)\,.\end{aligned} \tag{3}$$

All throughout this section the integration domain will be implied to be $U(d)$, and unless otherwise specified we consider W to be an operator acting on a Hilbert space $\mathcal{H}_w$ of dimension d. When $d=2^m$, as occurs for a Hilbert space of m qubit, we adopt the symbol $\boldsymbol{i}=(i_1,\dots i_m)$ to denote a bitstring of length m such that $i_1,i_2,\dots,i_m\in\{0,1\}$. Moreover, given two bitstrings $\boldsymbol{i}$ and $\boldsymbol{j}$ we define their concatenation as $\boldsymbol{i}\cdot\boldsymbol{j}=(i_1,\dots i_n,j_1,\dots j_n)$.

Operators in the computational basis of m qubits can be written as

$$W=\sum_{\boldsymbol{i},\boldsymbol{j}} w_{\boldsymbol{i},\boldsymbol{j}}|\boldsymbol{i}\rangle\langle\boldsymbol{j}|\,,\quad W^\dagger=\sum_{\boldsymbol{i}',\boldsymbol{j}'} w^*_{\boldsymbol{i}',\boldsymbol{j}'}|\boldsymbol{j}'\rangle\langle\boldsymbol{i}'|\,,\quad A=\sum_{\boldsymbol{k},\boldsymbol{l}} a_{\boldsymbol{k},\boldsymbol{l}}|\boldsymbol{k}\rangle\langle\boldsymbol{l}|\,,\quad B=\sum_{\boldsymbol{q},\boldsymbol{p}} b_{\boldsymbol{q},\boldsymbol{p}}|\boldsymbol{q}\rangle\langle\boldsymbol{p}|\,.$$

From the previous expressions we can derive the following lemmas.

Lemma 1. Let $\{W_y\}_{y\in Y}\subset U(d)$ form a unitary t-design with $t\geq 1$, and let $A,B:\mathcal{H}_w\to\mathcal{H}_w$ be arbitrary linear operators. Then

$$\frac{1}{|Y|}\sum_{y\in Y}\mathrm{Tr}\left[W_y A W_y^\dagger B\right]=\int d\mu(W)\mathrm{Tr}\left[WAW^\dagger B\right]=\frac{\mathrm{Tr}\left[A\right]\mathrm{Tr}\left[B\right]}{d}\,. \tag{4}$$

Proof. The first equality follows from the definition of a t-design. Note that $\mathrm{Tr}\left[WAW^\dagger B\right]$ can be written as

$$\mathrm{Tr}\left[WAW^\dagger B\right] = \sum_{\boldsymbol{i}_1,\boldsymbol{j}_1,\boldsymbol{i}'_1,\boldsymbol{j}'_1} a_{\boldsymbol{j}_1,\boldsymbol{j}'_1} b_{\boldsymbol{i}'_1,\boldsymbol{i}_1} w_{\boldsymbol{i}_1,\boldsymbol{j}_1} w^*_{\boldsymbol{i}'_1,\boldsymbol{j}'_1} .$$

Then, from (2), we have

$$\int d\mu(W)\mathrm{Tr}\left[WAW^\dagger B\right] = \frac{1}{d}\sum_{\boldsymbol{i}_1,\boldsymbol{j}_1} a_{\boldsymbol{j}_1,\boldsymbol{j}_1} b_{\boldsymbol{i}_1,\boldsymbol{i}_1} = \frac{\mathrm{Tr}\left[A\right]\mathrm{Tr}\left[B\right]}{d} .$$

□

Lemma 2. Let $\{W_y\}_{y\in Y} \subset U(d)$ form a unitary t-design with $t \geq 2$ and let $A, B, C, D : \mathcal{H}_w \to \mathcal{H}_w$ be arbitrary linear operators. Then

$$\begin{aligned}\frac{1}{|Y|}\sum_{y\in Y}\mathrm{Tr}[W_y A W_y^\dagger B W_y C W_y^\dagger D] = \int d\mu(W)\mathrm{Tr}[WAW^\dagger BWCW^\dagger D] \\ = \frac{1}{d^2-1}\left(\mathrm{Tr}[A]\mathrm{Tr}[C]\mathrm{Tr}[BD] + \mathrm{Tr}[AC]\mathrm{Tr}[B]\mathrm{Tr}[D]\right) \\ - \frac{1}{d(d^2-1)}\left(\mathrm{Tr}[AC]\mathrm{Tr}[BD] + \mathrm{Tr}[A]\mathrm{Tr}[B]\mathrm{Tr}[C]\mathrm{Tr}[D]\right) .\end{aligned} \tag{5}$$

Proof. The first equality follows from that fact that $\mathrm{Tr}[W_y A W_y^\dagger B W_y C W_y^\dagger D] \in P_{(2,2)}(W_y)$. By writting

$$\mathrm{Tr}[WAW^\dagger BWCW^\dagger D] = \sum_{\substack{\boldsymbol{i}_1,\boldsymbol{j}_1,\boldsymbol{i}'_1,\boldsymbol{j}'_1\\ \boldsymbol{i}_2,\boldsymbol{j}_2,\boldsymbol{i}'_2,\boldsymbol{j}'_2}} a_{\boldsymbol{j}_1,\boldsymbol{j}'_1} b_{\boldsymbol{i}'_1,\boldsymbol{i}_2} c_{\boldsymbol{j}_2,\boldsymbol{j}'_2} d_{\boldsymbol{i}'_2,\boldsymbol{i}_1} w_{\boldsymbol{i}_1,\boldsymbol{j}_1} w_{\boldsymbol{i}_2,\boldsymbol{j}_2} w^*_{\boldsymbol{i}'_1,\boldsymbol{j}'_1} w^*_{\boldsymbol{i}'_2,\boldsymbol{j}'_2} ,$$

we can use (3) to obtain

$$\begin{aligned}\int d\mu(W)\mathrm{Tr}[WAW^\dagger BWCW^\dagger D] = \frac{1}{d^2-1}\sum_{\boldsymbol{i}_1,\boldsymbol{j}_1,\boldsymbol{i}_2,\boldsymbol{j}_2}\left(a_{\boldsymbol{j}_1,\boldsymbol{j}_1} b_{\boldsymbol{i}_1,\boldsymbol{i}_2} c_{\boldsymbol{j}_2,\boldsymbol{j}_2} d_{\boldsymbol{i}_2,\boldsymbol{i}_1} + a_{\boldsymbol{j}_1,\boldsymbol{j}_2} b_{\boldsymbol{i}_2,\boldsymbol{i}_2} c_{\boldsymbol{j}_2,\boldsymbol{j}_1} d_{\boldsymbol{i}_1,\boldsymbol{i}_1}\right) \\ - \frac{1}{d(d^2-1)}\sum_{\boldsymbol{i}_1,\boldsymbol{j}_1,\boldsymbol{i}_2,\boldsymbol{j}_2}\left(a_{\boldsymbol{j}_1,\boldsymbol{j}_2} b_{\boldsymbol{i}_1,\boldsymbol{i}_2} c_{\boldsymbol{j}_2,\boldsymbol{j}_1} d_{\boldsymbol{i}_2,\boldsymbol{i}_1} + a_{\boldsymbol{j}_1,\boldsymbol{j}_1} b_{\boldsymbol{i}_2,\boldsymbol{i}_2} c_{\boldsymbol{j}_2,\boldsymbol{j}_2} d_{\boldsymbol{i}_1,\boldsymbol{i}_1}\right) \\ = \frac{1}{d^2-1}\left(\mathrm{Tr}[A]\mathrm{Tr}[C]\mathrm{Tr}[BD] + \mathrm{Tr}[AC]\mathrm{Tr}[B]\mathrm{Tr}[D]\right) \\ - \frac{1}{d(d^2-1)}\left(\mathrm{Tr}[AC]\mathrm{Tr}[BD] + \mathrm{Tr}[A]\mathrm{Tr}[B]\mathrm{Tr}[C]\mathrm{Tr}[D]\right) .\end{aligned} \tag{6}$$

□

Lemma 3. Let $\{W_y\}_{y\in Y} \subset U(d)$ form a unitary t-design with $t \geq 2$ and let $A, B, C, D : \mathcal{H}_w \to \mathcal{H}_w$ be arbitrary linear operators. Then

$$\begin{aligned}\frac{1}{|Y|}\sum_{y\in Y}\mathrm{Tr}[W_y A W_y^\dagger B]\mathrm{Tr}[W_y C W_y^\dagger D] = \int d\mu(W)\mathrm{Tr}[WAW^\dagger B]\mathrm{Tr}[WCW^\dagger D] \\ = \frac{1}{d^2-1}\left(\mathrm{Tr}[A]\mathrm{Tr}[B]\mathrm{Tr}[C]\mathrm{Tr}[D] + \mathrm{Tr}[AC]\mathrm{Tr}[BD]\right) \\ - \frac{1}{d(d^2-1)}\left(\mathrm{Tr}[AC]\mathrm{Tr}[B]\mathrm{Tr}[D] + \mathrm{Tr}[A]\mathrm{Tr}[C]\mathrm{Tr}[BD]\right) .\end{aligned}$$

Proof. The first equality follows from a reasoning similar to the one used in Lemma 2. By expressing

$$\mathrm{Tr}\left[WAW^\dagger B\right]\mathrm{Tr}\left[WCW^\dagger D\right] = \sum_{\boldsymbol{\alpha},\boldsymbol{\beta}}\mathrm{Tr}\left[WAW^\dagger B|\boldsymbol{\alpha}\rangle\langle\boldsymbol{\beta}|WCW^\dagger D|\boldsymbol{\beta}\rangle\langle\boldsymbol{\alpha}|\right] ,$$

we can employ (5) to obtain

$$\begin{aligned}\int d\mu(W)\mathrm{Tr}[WAW^\dagger B]\mathrm{Tr}[WCW^\dagger D] =&\sum_{\boldsymbol{\alpha},\boldsymbol{\beta}}\int d\mu(W)\mathrm{Tr}\left[WAW^\dagger B|\boldsymbol{\alpha}\rangle\langle\boldsymbol{\beta}|WCW^\dagger D|\boldsymbol{\beta}\rangle\langle\boldsymbol{\alpha}|\right]\\ =&\frac{1}{d^2-1}\sum_{\boldsymbol{\alpha},\boldsymbol{\beta}}\left(\mathrm{Tr}[A]\mathrm{Tr}[C]\langle\boldsymbol{\alpha}|B|\boldsymbol{\alpha}\rangle\langle\boldsymbol{\beta}|D|\boldsymbol{\beta}\rangle+\mathrm{Tr}[AC]\langle\boldsymbol{\beta}|B|\boldsymbol{\alpha}\rangle\langle\boldsymbol{\alpha}|D|\boldsymbol{\beta}\rangle\right)\\ &-\frac{1}{d(d^2-1)}\sum_{\boldsymbol{\alpha},\boldsymbol{\beta}}\left(\mathrm{Tr}[AC]\langle\boldsymbol{\alpha}|B|\boldsymbol{\alpha}\rangle\langle\boldsymbol{\beta}|D|\boldsymbol{\beta}\rangle+\mathrm{Tr}[A]\mathrm{Tr}[C]\langle\boldsymbol{\beta}|B|\boldsymbol{\alpha}\rangle\langle\boldsymbol{\alpha}|D|\boldsymbol{\beta}\rangle\right)\\ =&\frac{1}{d^2-1}\left(\mathrm{Tr}[A]\mathrm{Tr}[B]\mathrm{Tr}[C]\mathrm{Tr}[D]+\mathrm{Tr}[AC]\mathrm{Tr}[BD]\right)\\ &-\frac{1}{d(d^2-1)}\left(\mathrm{Tr}[AC]\mathrm{Tr}[B]\mathrm{Tr}[D]+\mathrm{Tr}[A]\mathrm{Tr}[C]\mathrm{Tr}[BD]\right).\end{aligned}$$

□

Lemma 4. Let $\mathcal{H}=\mathcal{H}_{\overline{w}}\otimes\mathcal{H}_w$ be a bipartite Hilbert space of dimension $d=d_{\overline{w}}d_w$, and let $\{W_y\}_{y\in Y}$ be a unitary t-design with $t\geq 1$ such that $W_y\in U(d_w)$ for all $y\in Y$. Then for arbitrary linear operators $A,B:\mathcal{H}\to\mathcal{H}$, we have

$$\int d\mu(W)(\mathbb{1}_{\overline{w}}\otimes W)A(\mathbb{1}_{\overline{w}}\otimes W^\dagger)B=\frac{\mathrm{Tr}_w[A]\otimes\mathbb{1}_w}{d_w}B\,, \tag{7}$$

and

$$\int d\mu(W)\mathrm{Tr}\left[(\mathbb{1}_{\overline{w}}\otimes W)A(\mathbb{1}_{\overline{w}}\otimes W^\dagger)B\right]=\frac{1}{d_w}\mathrm{Tr}\left[\mathrm{Tr}_w[A]\,\mathrm{Tr}_w[B]\right]. \tag{8}$$

Here we use the notation $\mathbb{1}_w$ to indicate the identity operator on subsystem $\mathcal{H}_w$, and we employ Tr_w to indicate the partial trace over $\mathcal{H}_w$.

Proof. First, note that

$$(\mathbb{1}_{\overline{w}}\otimes W)A(\mathbb{1}_{\overline{w}}\otimes W^\dagger)B=\sum_{\boldsymbol{i},\boldsymbol{j},\boldsymbol{i'},\boldsymbol{j'}}w_{\boldsymbol{i},\boldsymbol{j}}w^*_{\boldsymbol{i'},\boldsymbol{j'}}(\mathbb{1}_{\overline{w}}\otimes|\boldsymbol{i}\rangle\langle\boldsymbol{j}|)A(\mathbb{1}_{\overline{w}}\otimes|\boldsymbol{j'}\rangle\langle\boldsymbol{i'}|)B\,,$$

by using (2) we have

$$\begin{aligned}\int d\mu(W)(\mathbb{1}_{\overline{w}}\otimes W)A(\mathbb{1}_{\overline{w}}\otimes W^\dagger)B=&\sum_{\boldsymbol{i},\boldsymbol{j},\boldsymbol{i'},\boldsymbol{j'}}\int d\mu(W)w_{\boldsymbol{i},\boldsymbol{j}}w^*_{\boldsymbol{i'},\boldsymbol{j'}}(\mathbb{1}_{\overline{w}}\otimes|\boldsymbol{i}\rangle\langle\boldsymbol{j}|)A(\mathbb{1}_{\overline{w}}\otimes|\boldsymbol{j'}\rangle\langle\boldsymbol{i'}|)B\\ =&\frac{1}{d_w}\sum_{\boldsymbol{i},\boldsymbol{j}}(\mathbb{1}_{\overline{w}}\otimes|\boldsymbol{i}\rangle\langle\boldsymbol{j}|)A(\mathbb{1}_{\overline{w}}\otimes|\boldsymbol{j}\rangle\langle\boldsymbol{i}|)B\\ =&\frac{1}{d_w}\left(\mathrm{Tr}_w[A]\otimes\mathbb{1}_w\right)B\,.\end{aligned}$$

Finally we can also obtain

$$\int d\mu(W)\mathrm{Tr}\left[(\mathbb{1}_{\overline{w}}\otimes W)A(\mathbb{1}_{\overline{w}}\otimes W^\dagger)B\right]=\frac{1}{d_w}\mathrm{Tr}\left[\mathrm{Tr}_w[A]\,\mathrm{Tr}_w[B]\right].$$

□

Lemma 5. If $\mathcal{H}=\mathcal{H}_{\overline{w}}\otimes\mathcal{H}_w$ is a bipartite Hilbert space of dimension $d=d_{\overline{w}}d_w$ ($d=2^n$, and $\overline{d}=2^{n'}$), and if $A,B:\mathcal{H}\to\mathcal{H}$ are arbitrary linear operators, then for any linear operator $W:\mathcal{H}_w\to\mathcal{H}_w$ we have

$$\mathrm{Tr}\left[(\mathbb{1}_{\overline{w}}\otimes W)A(\mathbb{1}_{\overline{w}}\otimes W^\dagger)B\right]=\sum_{\boldsymbol{p},\boldsymbol{q}}\mathrm{Tr}\left[WA_{\boldsymbol{qp}}W^\dagger B_{\boldsymbol{pq}}\right], \tag{9}$$

where the summation runs over all bitstrings of length n', and where

$$A_{\boldsymbol{qp}}=\mathrm{Tr}_{\overline{w}}[(|\boldsymbol{p}\rangle\langle\boldsymbol{q}|\otimes\mathbb{1}_w)A]\,,\quad B_{\boldsymbol{pq}}=\mathrm{Tr}_{\overline{w}}[(|\boldsymbol{q}\rangle\langle\boldsymbol{p}|\otimes\mathbb{1}_w)B]\,. \tag{10}$$

Proof. By expanding the operators in the computational basis

$$\mathbb{1}_{\overline{w}} \otimes W = \sum_{\boldsymbol{p},\boldsymbol{i},\boldsymbol{j}} w_{\boldsymbol{i},\boldsymbol{j}} |\boldsymbol{p}\rangle\langle\boldsymbol{p}| \otimes |\boldsymbol{i}\rangle\langle\boldsymbol{j}| \,, \qquad \mathbb{1}_{\overline{w}} \otimes W^\dagger = \sum_{\boldsymbol{q},\boldsymbol{i}',\boldsymbol{j}'} w^*_{\boldsymbol{i}',\boldsymbol{j}'} |\boldsymbol{q}\rangle\langle\boldsymbol{q}| \otimes |\boldsymbol{j}'\rangle\langle\boldsymbol{i}'| \,, \tag{11}$$

$$A = \sum_{\substack{\boldsymbol{k}_1,\boldsymbol{k}_2\\ \boldsymbol{l}_1,\boldsymbol{l}_2}} a_{\boldsymbol{k}_1\cdot\boldsymbol{k}_2,\boldsymbol{l}_1\cdot\boldsymbol{l}_2} |\boldsymbol{k}_1\rangle\langle\boldsymbol{l}_1| \otimes |\boldsymbol{k}_2\rangle\langle\boldsymbol{l}_2| \,, \quad B = \sum_{\substack{\boldsymbol{p}_1,\boldsymbol{p}_2\\ \boldsymbol{q}_1,\boldsymbol{q}_2}} b_{\boldsymbol{p}_1\cdot\boldsymbol{p}_2,\boldsymbol{q}_1\cdot\boldsymbol{q}_2} |\boldsymbol{p}_1\rangle\langle\boldsymbol{q}_1| \otimes |\boldsymbol{p}_2\rangle\langle\boldsymbol{q}_2| \,, \tag{12}$$

we have

$$\mathrm{Tr}\left[(\mathbb{1}_{\overline{w}} \otimes W)A(\mathbb{1}_{\overline{w}} \otimes W^\dagger)B\right] = \sum_{\substack{\boldsymbol{i},\boldsymbol{j},\boldsymbol{i}',\boldsymbol{j}'\\ \boldsymbol{p},\boldsymbol{q}}} w_{\boldsymbol{i},\boldsymbol{j}} a_{\boldsymbol{p}\cdot\boldsymbol{j},\boldsymbol{q}\cdot\boldsymbol{i}'} w_{\boldsymbol{i}',\boldsymbol{j}'} b_{\boldsymbol{q}\cdot\boldsymbol{j}',\boldsymbol{p}\cdot\boldsymbol{i}} = \sum_{\boldsymbol{p},\boldsymbol{q}} \mathrm{Tr}\left[W A_{\boldsymbol{q}\boldsymbol{p}} W^\dagger B_{\boldsymbol{p}\boldsymbol{q}}\right] \,. \tag{13}$$

□

Lemma 6. Let $\mathcal{H} = \mathcal{H}_1 \otimes \mathcal{H}_2 \otimes \mathcal{H}_3 \otimes \mathcal{H}_4$ be a Hilbert space of dimension $d \cdot d \cdot d \cdot d = d^4$, and let $W : \mathcal{H}_1 \otimes \mathcal{H}_2 \to \mathcal{H}_1 \otimes \mathcal{H}_2$ be a t-design with $t \geqslant 2$. For arbitrary linear operators $A, A' : \mathcal{H} \to \mathcal{H}$ we define

$$\Omega_1 = (W \otimes \mathbb{1}_3 \otimes \mathbb{1}_4)A(W^\dagger \otimes \mathbb{1}_3 \otimes \mathbb{1}_4)(|\boldsymbol{p}\rangle\langle\boldsymbol{q}| \otimes \mathbb{1}_2 \otimes \mathbb{1}_3 \otimes \mathbb{1}_4) \,, \;\; \Omega_2 = (W \otimes \mathbb{1}_3 \otimes \mathbb{1}_4)A(W^\dagger \otimes \mathbb{1}_3 \otimes \mathbb{1}_4) \,, \tag{14}$$

$$\Omega_1' = (W \otimes \mathbb{1}_3 \otimes \mathbb{1}_4)A'(W^\dagger \otimes \mathbb{1}_3 \otimes \mathbb{1}_4)(|\boldsymbol{p}'\rangle\langle\boldsymbol{q}'| \otimes \mathbb{1}_2 \otimes \mathbb{1}_3 \otimes \mathbb{1}_4) \,, \;\; \Omega_2' = (W \otimes \mathbb{1}_3 \otimes \mathbb{1}_4)A'(W^\dagger \otimes \mathbb{1}_3 \otimes \mathbb{1}_4) \,, \tag{15}$$

where $\boldsymbol{p}$, $\boldsymbol{q}$, $\boldsymbol{p}'$, and $\boldsymbol{q}'$ are bitstrings of length d.

Let us first consider the quantity

$$\Delta\Omega^{(1)}_{jkl} = \mathrm{Tr}_{kl}\left[\mathrm{Tr}_{1j}[\Omega_1]\mathrm{Tr}_{1j}[\Omega_1']\right] - \frac{\mathrm{Tr}_{\mathrm{L}}[\mathrm{Tr}_{1jk}[\Omega_1]_{1jk}\mathrm{Tr}[\Omega_1']]}{d_k} \,, \tag{16}$$

where Tr_j indicates the partial trace over $\mathcal{H}_j$, and where $\mathrm{Tr}_{jk} := \mathrm{Tr}_j\mathrm{Tr}_k$. Here the indexes j, k, and l define Hilbert spaces such that $\mathcal{H}_j \otimes \mathcal{H}_k \otimes \mathcal{H}_{\mathrm{L}} = \mathcal{H}_2 \otimes \mathcal{H}_3 \otimes \mathcal{H}_4$. In addition, let us define $\mathcal{H}_j = \mathcal{H}_0 := \{\mathbf{0}\}$, in which case for any operator O we also define the partial trace over $\mathcal{H}_0$ as $\mathrm{Tr}_0[O] := O$, and $\mathrm{Tr}_{k0}[O] := \mathrm{Tr}_k[O]$. From the previous definition, we then have that $\mathcal{H}_k$ can be chosen from the set $\{\mathcal{H}_2, \mathcal{H}_3, \mathcal{H}_2 \otimes \mathcal{H}_3, \mathcal{H}_3 \otimes \mathcal{H}_4\}$, and for any such choice the following equality always holds

$$\begin{aligned}
\int d\mu(W)\Delta\Omega^{(1)}_{jkl} =& \frac{t_1^{(1)}\delta_{\boldsymbol{p}\boldsymbol{q}'}\delta_{\boldsymbol{p}'\boldsymbol{q}}}{d^4-1}\left(\mathrm{Tr}_{123x}[\mathrm{Tr}_y[A]\mathrm{Tr}_y[A']] - \frac{\mathrm{Tr}_{3x}[\mathrm{Tr}_{12y}[A]\mathrm{Tr}_{12y}[A']]}{d^2}\right)\\
&+ \frac{t_2^{(1)}\delta_{\boldsymbol{p}\boldsymbol{q}}\delta_{\boldsymbol{p}'\boldsymbol{q}'}}{d^4-1}\left(\mathrm{Tr}_{3x}[\mathrm{Tr}_{12y}[A]\mathrm{Tr}_{12y}[A']] - \frac{\mathrm{Tr}_x[\mathrm{Tr}_{123y}[A]\mathrm{Tr}_{123y}[A']]}{d'}\right)\\
&- \frac{t_3^{(1)}\delta_{\boldsymbol{p}\boldsymbol{q}'}\delta_{\boldsymbol{p}'\boldsymbol{q}}}{d^4-1}\left(\mathrm{Tr}_{12x}[\mathrm{Tr}_{3y}[A]\mathrm{Tr}_{3y}[A']] - \frac{\mathrm{Tr}_x[\mathrm{Tr}_{123y}[A]\mathrm{Tr}_{123y}[A']]}{d^2}\right)\\
&- \frac{t_4^{(1)}\delta_{\boldsymbol{p}\boldsymbol{q}}\delta_{\boldsymbol{p}'\boldsymbol{q}'}}{d^4-1}\left(\mathrm{Tr}_{123x}[\mathrm{Tr}_y[A]\mathrm{Tr}_y[A']] - \frac{\mathrm{Tr}_{12x}[\mathrm{Tr}_{3y}[A]\mathrm{Tr}_{3y}[A']]}{d'}\right)
\end{aligned} \tag{17}$$

where $d' = d^2$ if $\mathcal{H}_4 \leqslant \mathcal{H}_k$, and $d' = d$ otherwise. Here we employ the notation $A \leqslant B$ to indicate that A is a subspace of B. Note that $\mathcal{H}_y = \mathcal{H}_4$, $\mathcal{H}_x = \mathcal{H}_0$ if $\mathcal{H}_4 \leqslant \mathcal{H}_j$, and $\mathcal{H}_y = \mathcal{H}_0$, $\mathcal{H}_x = \mathcal{H}_4$ otherwise. In addition, we have

$$t_1^{(1)} = \begin{cases} d & \text{if } \mathcal{H}_2 \leqslant \mathcal{H}_j \\ 0 & \text{if } \mathcal{H}_k = \mathcal{H}_2 \\ d^2 & \text{otherwise}\end{cases}, \quad t_2^{(1)} = \begin{cases} d^2 & \text{if } \mathcal{H}_2 \leqslant \mathcal{H}_j \\ 0 & \text{if } \mathcal{H}_k = \mathcal{H}_2 \\ d & \text{otherwise}\end{cases}, \quad t_4^{(1)} = \begin{cases} 1 & \text{if } \mathcal{H}_2 \leqslant \mathcal{H}_j, \text{ and } \mathcal{H}_3 \in \mathcal{H}_k \\ 0 & \text{if } \mathcal{H}_k = \mathcal{H}_1 \\ \frac{1}{d} & \text{otherwise}\end{cases} \tag{18}$$

$$t_3^{(1)} = \begin{cases} d & \text{if } \mathcal{H}_k = \mathcal{H}_3, \text{ and } \mathcal{H}_2 \leqslant \mathcal{H}_{\mathrm{L}} \\ 1-d^2 & \text{if } \mathcal{H}_k = \mathcal{H}_2 \\ \frac{1}{d} & \text{if } \mathcal{H}_k = \mathcal{H}_2 \otimes \mathcal{H}_3, \text{ or if } \mathcal{H}_k = \mathcal{H}_3 \otimes \mathcal{H}_4, \text{ and } \mathcal{H}_j = \mathcal{H}_2 \\ 1 & \text{otherwise}\end{cases}, \tag{19}$$

where we can verify that for all 12 possible cases that $\sum_{k=1}^4 |\frac{t_k^{(1)}}{(d^4-1)}| \leqslant 1$.

Second, let us consider the quantity

$$\Delta\Omega^{(2)}_{jkl} = \mathrm{Tr}_{kl}\left[\mathrm{Tr}_{1j}[\Omega_2]\mathrm{Tr}_{1j}[\Omega_2']\right] - \frac{\mathrm{Tr}_{\mathrm{L}}[\mathrm{Tr}_{12j}[\Omega_2]_{1jk}\mathrm{Tr}[\Omega_2']]}{d_k} \,. \tag{20}$$

Then, for $\mathcal{H}_k \in \{\mathcal{H}_2, \mathcal{H}_3, \mathcal{H}_2 \otimes \mathcal{H}_3, \mathcal{H}_3 \otimes \mathcal{H}_4\}$, the following equality always holds

$$\begin{aligned}\int d\mu(W)\Delta\Omega^{(2)}_{jkl} =& \frac{t_1^{(2)}}{d^2+1}\left(\mathrm{Tr}_{123x}[\mathrm{Tr}_y[A]\mathrm{Tr}_y[A']] - \frac{\mathrm{Tr}_{3x}[\mathrm{Tr}_{12y}[A]\mathrm{Tr}_{12y}[A']]}{d^2}\right)\\ &+ \frac{t_2^{(2)}}{d^2+1}\left(\mathrm{Tr}_{3x}[\mathrm{Tr}_{12y}[A]\mathrm{Tr}_{12y}[A']] - \frac{\mathrm{Tr}_x[\mathrm{Tr}_{123y}[A]\mathrm{Tr}_{123y}[A']]}{d'}\right)\\ &- \frac{t_3^{(2)}}{d^2+1}\left(\mathrm{Tr}_{12x}[\mathrm{Tr}_{3y}[A]\mathrm{Tr}_{3y}[A']] - \frac{\mathrm{Tr}_x[\mathrm{Tr}_{123y}[A]\mathrm{Tr}_{123y}[A']]}{d^2}\right),\end{aligned} \tag{21}$$

where $d' = d^2$ if $\mathcal{H}_4 \leqslant \mathcal{H}_k$, and $d' = d$ otherwise. Similarly, $\mathcal{H}_y = \mathcal{H}_4$, $\mathcal{H}_x = \mathcal{H}_0$ if $\mathcal{H}_4 \leqslant \mathcal{H}_j$, and $\mathcal{H}_y = \mathcal{H}_0$, $\mathcal{H}_x = \mathcal{H}_4$ otherwise. In addition, we now have

$$t_1^{(2)} = \begin{cases} 0 & \text{if } \mathcal{H}_2 \leqslant \mathcal{H}_j \\ d & \text{otherwise}\end{cases}, \quad t_2^{(2)} = \begin{cases} 0 & \text{if } \mathcal{H}_2 \leqslant \mathcal{H}_j, \text{ or } \mathcal{H}_k = \mathcal{H}_2 \\ \frac{d^2+1}{d} & \text{otherwise}\end{cases}, \quad t_3^{(2)} = \begin{cases} 1 & \text{if } \mathcal{H}_2 \leqslant \mathcal{H}_\mathrm{L}, \text{ and } \mathcal{H}_k = \mathcal{H}_3 \\ \frac{1}{d} & \text{if } \mathcal{H}_\mathrm{L} = \mathcal{H}_2, \text{ and } \mathcal{H}_k = \mathcal{H}_3 \otimes \mathcal{H}_4 \\ 0 & \text{otherwise}\end{cases}, \tag{22}$$

where we can verify that for all 9 nontrivial cases $\sum_{k=1}^3 |\frac{t_k^{(2)}}{d^2+1}| \leqslant 1$.

Proof. The proof of Eqs. (17), and (21) can be obtain by explicitly integrating each term via (3). □

In particular, let us consider from Eq. (17) the following case which will be relevant for our proofs.

$$\begin{aligned}\int d\mu(W)\left(\mathrm{Tr}_{34}\left[\mathrm{Tr}_{12}[\Omega_1]\mathrm{Tr}_{12}[\Omega_1']\right] - \frac{\mathrm{Tr}[\Omega_1]\mathrm{Tr}[\Omega_1']}{d^2}\right) =& \frac{d^2\delta_{\boldsymbol{p}_2\boldsymbol{q}_2'}\delta_{\boldsymbol{p}_2'\boldsymbol{q}_2}}{d^4-1}\left(\mathrm{Tr}_{234}[\mathrm{Tr}_1[A]\mathrm{Tr}_1[A']] - \frac{\mathrm{Tr}_4[\mathrm{Tr}_{123}[A]\mathrm{Tr}_{123}[A']]}{d^2}\right)\\ &+ \frac{d\delta_{\boldsymbol{p}_2\boldsymbol{q}_2}\delta_{\boldsymbol{p}_2'\boldsymbol{q}_2'}}{d^4-1}\left(\mathrm{Tr}_4[\mathrm{Tr}_{123}[A]\mathrm{Tr}_{123}[A']] - \frac{\mathrm{Tr}[A]\mathrm{Tr}[A']}{d}\right)\\ &- \frac{\delta_{\boldsymbol{p}_2\boldsymbol{q}_2'}\delta_{\boldsymbol{p}_2'\boldsymbol{q}_2}}{d(d^4-1)}\left(\mathrm{Tr}_{23}[\mathrm{Tr}_{14}[A]\mathrm{Tr}_{14}[A']] - \frac{\mathrm{Tr}[A]\mathrm{Tr}[A']}{d^2}\right)\\ &- \frac{\delta_{\boldsymbol{p}_2\boldsymbol{q}_2}\delta_{\boldsymbol{p}_2'\boldsymbol{q}_2'}}{d(d^4-1)}\left(\mathrm{Tr}_{234}[\mathrm{Tr}_1[A]\mathrm{Tr}_1[A']] - \frac{\mathrm{Tr}_{23}[\mathrm{Tr}_{14}[A]\mathrm{Tr}_{14}[A']]}{d}\right).\end{aligned} \tag{23}$$

Lemma 7. Let $\mathcal{H} = \mathcal{H}_1 \otimes \mathcal{H}_2 \otimes \mathcal{H}_3$ be a tripartite Hilbert space of dimension $d = d_1 d_2 d_3$, and let $O_1 = O_\mathrm{A} \otimes \mathbb{1}_2 \otimes \mathbb{1}_3$, and $O_2 = \mathbb{1}_1 \otimes O_\mathrm{B} \otimes \mathbb{1}_3$ be linear operators on S. Here $\mathbb{1}_i$ indicates the identity over subsystem S_i, so that O_1 and O_2 have no overlapping support. Then for any linear operators $O_\mathrm{A} : S_1 \to S_1$, and $O_\mathrm{B} : S_2 \to S_2$ we have

$$\mathrm{Tr}_{jk}\left[\mathrm{Tr}_i\left[O_1\right]\mathrm{Tr}_i\left[O_2\right]\right] - \frac{\mathrm{Tr}_k\left[\mathrm{Tr}_{ij}\left[O_1\right]\mathrm{Tr}_{ij}\left[O_2\right]\right]}{d_j} = 0\,, \tag{24}$$

where Tr_i indicates the partial trace over $\mathcal{H}_i$, $\mathrm{Tr}_{ij} := \mathrm{Tr}_i\mathrm{Tr}_j$, and where we defined $\mathcal{H}_i \otimes \mathcal{H}_j \otimes \mathcal{H}_k = \mathcal{H}$ such that $\mathcal{H}_j = \mathcal{H}_1, \mathcal{H}_2$ or $\mathcal{H}_3$. Moreover, one can always choose $\mathcal{H}_i = \mathcal{H}_0 := \{\mathbf{0}\}$ (or $\mathcal{H}_k = \mathcal{H}_0$), in which case we define the partial trace over $\mathcal{H}_0$ as $\mathrm{Tr}_0\left[O\right] := O$, and $\mathrm{Tr}_{j0}\left[O\right] := \mathrm{Tr}_j\left[O\right]$.

Proof. Let us show how this equality holds for the specific case when $\mathcal{H}_i = \{\mathbf{0}\}$, $\mathcal{H}_j = \mathcal{H}_2$, and $\mathcal{H}_k = \mathcal{H}_1 \otimes \mathcal{H}_3$, and let us remark that all remaining cases follow similarly We have

$$\mathrm{Tr}_0\left[O_1\right]\mathrm{Tr}_0\left[O_2\right] = O_\mathrm{A} \otimes O_\mathrm{B} \otimes \mathbb{1}_3\,, \quad \mathrm{Tr}_2\left[O_1\right] = d_2 O_\mathrm{A} \otimes \mathbb{1}_3\,, \quad \mathrm{Tr}_2\left[O_2\right] = \mathrm{Tr}_2\left[O_\mathrm{B}\right]\mathbb{1}_1 \otimes \mathbb{1}_3\,, \tag{25}$$

and Eq. (24) becomes

$$\mathrm{Tr}_{123}\left[O_1 O_2\right] - \frac{\mathrm{Tr}_{13}\left[\mathrm{Tr}_2\left[O_1\right]\mathrm{Tr}_2\left[O_2\right]\right]}{d_2} = \mathrm{Tr}_{123}\left[O_\mathrm{A} \otimes O_\mathrm{B} \otimes \mathbb{1}_3\right] - \frac{d_2\mathrm{Tr}_2\left[O_\mathrm{B}\right]\mathrm{Tr}_{13}\left[O_\mathrm{A} \otimes \mathbb{1}_3\right]}{d_2} \tag{26}$$

$$= d_3\mathrm{Tr}_1\left[O_\mathrm{A}\right]\mathrm{Tr}_2\left[O_\mathrm{B}\right] - \frac{d_2 d_3\mathrm{Tr}_1\left[O_\mathrm{A}\right]\mathrm{Tr}_2\left[O_\mathrm{B}\right]}{d_2} \tag{27}$$

$$= 0\,. \tag{28}$$

□

Supplementary Note 2: Proof of Proposition 1

Here we provide a proof for Proposition 1, which we recall for convenience:

Proposition 1. Let θ^j be uniformly distributed on $[-\pi, \pi]$ $\forall j$. For any $\delta \in (0,1)$, the probability that $C_{\rm G} \leq \delta$ satisfies

$$\Pr\{C_{\rm G} \leq \delta\} \leq (1-\delta)^{-1} \left(\frac{1}{2}\right)^n . \tag{29}$$

For any $\delta \in [\frac{1}{2}, 1]$, the probability that $C_{\rm L} \leq \delta$ satisfies

$$\Pr\{C_{\rm L} \leq \delta\} \geq \frac{(2\delta-1)^2}{\frac{1}{2n} + (2\delta-1)^2} \underset{n\to\infty}{\longrightarrow} 1 . \tag{30}$$

Proposition 1 formalizes the narrow gorge phenomenon shown in Fig. 2 of the main text for the warm-up example. Specifically, it bounds the volume of parameter space that is trainable in the sense that the cost deviates from its maximum value of one. As a consequence, one finds that the probability that the global cost function is less than 1 vanishes exponentially as $n \to \infty$, whereas for the local cost function there are neighbohoods of the minimum that have constant probability as $n \to \infty$. In the following proof, it is helpful to define the trainable region for the global and local cost as $A^G_\delta := \{\theta : C_G(\theta) \leqslant \delta\}$ and $A^L_\delta := \{\theta : C_{\rm L}(\theta) \leqslant \delta\}$.

Proof. Let us first consider the global cost function

$$C_G = {\rm Tr}[O_G V(\boldsymbol{\theta})|\mathbf{0}\rangle\langle\mathbf{0}|V(\boldsymbol{\theta})^\dagger] , \tag{31}$$

with $O_G = \mathbb{1} - |\mathbf{0}\rangle\langle\mathbf{0}|$, and with $V(\boldsymbol{\theta}) = \bigotimes_{j=1}^n e^{-i\theta^j \sigma_x^{(j)}/2}$. The probability of the region A^G_δ can be bounded from above by defining the random variable $X = \prod_{j=1}^n \cos^2 \frac{\theta_j}{2} = 1 - C_{\rm G}$ and noting that $(1-\delta)P\{X \geq 1-\delta\} \leq E(X)$. Then

$$\begin{aligned} \Pr(A^G_\delta) &= P\{\prod_{j=1}^n \cos^2 \frac{\theta^j}{2} \geq 1-\delta\} \\ &\leq (1-\delta)^{-1} E\left(\prod_{j=1}^n \cos^2 \frac{\theta^j}{2}\right) \\ &= (1-\delta)^{-1} \left(\frac{1}{2}\right)^n \end{aligned} \tag{32}$$

Therefore, for any $\delta \in (0,1)$, the probability that $C_{\rm G} \leq \delta$ goes to zero exponentially with n.

Now consider the local cost function $C_{\rm L}$ given by

$$C_{\rm L} = {\rm Tr}\left[O_{\rm L} V(\boldsymbol{\theta})|\mathbf{0}\rangle\langle\mathbf{0}|V(\boldsymbol{\theta})^\dagger\right] , \quad \text{with} \quad O_{\rm L} = \mathbb{1} - \frac{1}{n}\sum_{j=1}^n |0\rangle\langle 0|_j \otimes \mathbb{1}_{\bar{j}} , \tag{33}$$

and where $\mathbb{1}_{\bar{j}}$ is the identity on all qubits except qubit j. To show the dependence of the result on the range of local cost function values, we write a parametrized local cost function $C_{\rm L}(;\lambda) := 1 - \frac{1}{\lambda n}\sum_{j=1}^n \cos^2 \frac{\theta_j}{2}$ and define $A^L_\delta := \{\theta : C_{\rm L}(\theta;\lambda) \leqslant \delta\}$. $C_{\rm L}$ in the main text is obtained for $\lambda = 1$. The parameter λ is introduced to show that the range of validity of the inequality is dependent on the cost function. For δ in the interval $\delta \in (1-(2\lambda)^{-1}, 1]$,

$$\Pr\left(A^L_\delta\right) = \Pr\left(\left\{\theta : \frac{1}{\lambda n}\sum_{j=1}^n \cos^2 \frac{\theta^j}{2} \geq 1-\delta\right\}\right) \geqslant \frac{(1-2\lambda(1-\delta))^2}{\frac{1}{2n} + (1-2\lambda(1-\delta))^2} \tag{34}$$

which tends to 1 as $n \to \infty$. The inequality follows from the Paley-Zygmund inequality [4] in the form

$$P(X \geq rE(X)) \geqslant \frac{(1-r)^2 E(X)^2}{{\rm Var}X + (1-r)^2 E(X)^2} \tag{35}$$

which holds for random variables $X \geq 0$ and scalar r with $0 \leqslant r \leqslant 1$. In particular, (34) follows by taking $X = \frac{1}{n\lambda}\sum_{j=1}^n \cos^2 \frac{\theta^j}{2}$ and $r = 2\lambda(1-\delta)$, so that r varies from 1 to 0 as δ varies from $1-(2\lambda)^{-1}$ to 1. □

Supplementary Note 3: Proof of Proposition 2

Let us first recall that in the main text we analyze cost functions C which can be expressed as the expectation value of a given observable O as

$$C = \mathrm{Tr}\left[OV(\boldsymbol{\theta})\rho V^{\dagger}(\boldsymbol{\theta})\right] , \tag{36}$$

where $V(\boldsymbol{\theta})$ is a parametrized quantum gate sequence and ρ is a general input mixed quantum state on n qubits. Here we provide a proof for Proposition 2, which we recall for convenience:

Proposition 2. The average of the partial derivative of any cost function of the form (36) with respect to a parameter θ^{ν} in a block W of the ansatz $V(\boldsymbol{\theta})$ is

$$\langle \partial_{\nu} C \rangle_V = 0 \,, \tag{37}$$

provided that either W_{A} or W_{B} form a 1-design.

As discussed in the main text, we employ an Alternating Layered Ansatz, where each layer is composed of m-qubits gates or "blocks". In particular, each block $W_{kl}(\boldsymbol{\theta}_{kl})$ in $V(\boldsymbol{\theta})$ can be written as a product of ζ_{kl} independent gates from a gate alphabet $\mathcal{A} = \{G_{\mu}(\theta)\}$ as

$$W_{kl}(\boldsymbol{\theta}_{kl}) = G_{\zeta_{kl}}(\theta_{kl}^{\zeta_{kl}}) \dots G_{\nu}(\theta_{kl}^{\nu}) \dots G_1(\theta_{kl}^{1}) \,, \tag{38}$$

where θ_{kl}^{ν} are continuous parameter, and where $G_{\nu}(\theta_{kl}^{\nu}) = R_{\nu}(\theta_{kl}^{\nu})Q_{\nu}$ with Q_{ν} an unparametrized gate, and $R_{\nu}(\theta_{kl}^{\nu}) = e^{-i\theta_{kl}^{\nu}\sigma_{\nu}/2}$ such that σ_{ν} is a Pauli operator.

Consider now a block $W_{kl}(\boldsymbol{\theta}_{kl})$ in the l-th layer of the ansatz. For the rest of this Supplementary Information we simply use the notation W when referring to this particular block. First, let S_w denote the m-qubit subsystem that contains the qubits W acts on, and let $S_{\overline{w}}$ be the $(n-m)$ subsystem on which W acts trivially, with $\mathcal{H}_w$, and $\mathcal{H}_{\overline{w}}$ their respective associated Hilbert spaces. Then, consider a given trainable parameter θ^{ν} in W, such that we can express $W = W_{\mathrm{B}}W_{\mathrm{A}}$, with

$$W_{\mathrm{B}} = \prod_{\mu=1}^{\nu-1} G_{\mu}(\theta^{\mu}) \,, \quad \text{and} \quad W_{\mathrm{A}} = \prod_{\mu=\nu}^{\zeta} G_{\mu}(\theta^{\mu}) \,. \tag{39}$$

Then, we recall that we have defined the forward light-cone $\mathcal{L}$ of W as all gates with at least one input qubit causally connected to the output qubits of W. We can then define $S_{\mathcal{L}}$ as the subsystem of all qubits in $\mathcal{L}$, and $\mathcal{H}_{\mathcal{L}}$ as its associated Hilbert space. Without loss of generality, the trainable gate sequence can be expressed as

$$V(\boldsymbol{\theta}) = V_{\mathrm{R}}(\mathbb{1}_{\overline{w}} \otimes W)V_{\mathrm{L}} \,, \tag{40}$$

where $\mathbb{1}_{\overline{w}}$ indicates the identity in $\mathcal{H}_{\overline{w}}$, and where we assume without loss of generality that V_{R} contains the gates in $\mathcal{L}$ and all the blocks W_{kL} in the last layer of $V(\boldsymbol{\theta})$.

Proof. The partial derivative of W with respect to the angle θ_{ν} is given by

$$\partial_{\nu} W = W_{\mathrm{A}} \left(-\frac{i}{2}\sigma_{\nu}\right) W_{\mathrm{B}} \,, \tag{41}$$

where here σ_{ν} is an operator $\sigma_{\nu} : \mathcal{H}_w \to \mathcal{H}_w$, which acts non-trivially on a qubit given qubit j in $\mathcal{H}_w$, i.e., $\sigma_{\nu} := (\sigma_{\nu})_j \otimes \mathbb{1}_{\overline{j}}$. Hence, by means of Eqs. (41) and (40) we have

$$\begin{aligned}
\partial_{\nu} C =& \mathrm{Tr}\left[O\left(\partial_{\nu} V(\boldsymbol{\theta})\right)\rho V^{\dagger}(\boldsymbol{\theta}) + V(\boldsymbol{\theta})\rho\left(\partial_{\nu} V^{\dagger}(\boldsymbol{\theta})\right)\right] \\
=& \mathrm{Tr}\left[OV_{\mathrm{R}}\left(\mathbb{1}_{\overline{w}} \otimes W_{\mathrm{A}}\right)\left(\mathbb{1}_{\overline{w}} \otimes \left(-\frac{i}{2}\sigma_{\nu}\right)\right)\left(\mathbb{1}_{\overline{w}} \otimes W_{\mathrm{B}}\right)V_{\mathrm{L}}\rho V_{\mathrm{L}}^{\dagger}\left(\mathbb{1}_{\overline{w}} \otimes W_{\mathrm{B}}^{\dagger} W_{\mathrm{A}}^{\dagger}\right)V_{\mathrm{R}}^{\dagger}\right] \\
&+ \mathrm{Tr}\left[OV_{\mathrm{R}}\left(\mathbb{1}_{\overline{w}} \otimes W_{\mathrm{A}} W_{\mathrm{B}}\right)V_{\mathrm{L}}\rho V_{\mathrm{L}}^{\dagger}\left(\mathbb{1}_{\overline{w}} \otimes W_{\mathrm{B}}^{\dagger}\right)\left(\mathbb{1}_{\overline{w}} \otimes \left(+\frac{i}{2}\sigma_{\nu}\right)\right)\left(\mathbb{1}_{\overline{w}} \otimes W_{\mathrm{A}}^{\dagger}\right)V_{\mathrm{R}}^{\dagger}\right] .
\end{aligned}$$

Which can be simplified as

$$\partial_{\nu} C = \frac{i}{2}\mathrm{Tr}\left[\left(\mathbb{1}_{\overline{w}} \otimes W_{\mathrm{B}}\right)V_{\mathrm{L}}\rho V_{\mathrm{L}}^{\dagger}\left(\mathbb{1}_{\overline{w}} \otimes W_{\mathrm{B}}^{\dagger}\right)\left[\mathbb{1}_{\overline{w}} \otimes \sigma_{\nu}, \left(\mathbb{1}_{\overline{w}} \otimes W_{\mathrm{A}}^{\dagger}\right)V_{\mathrm{R}}^{\dagger} O V_{\mathrm{R}}\left(\mathbb{1}_{\overline{w}} \otimes W_{\mathrm{A}}\right)\right]\right] , \tag{42}$$

or equivalently, as

$$\partial_\nu C = -\frac{i}{2}\mathrm{Tr}\left[\left(\mathbb{1}_{\overline{w}}\otimes W_{\mathrm{A}}^\dagger\right)V_{\mathrm{R}}^\dagger O V_{\mathrm{R}}\left(\mathbb{1}_{\overline{w}}\otimes W_{\mathrm{A}}\right)\left[\mathbb{1}_{\overline{w}}\otimes\sigma_\nu,\left(\mathbb{1}_{\overline{w}}\otimes W_{\mathrm{B}}\right)V_{\mathrm{L}}\rho V_{\mathrm{L}}^\dagger\left(\mathbb{1}_{\overline{w}}\otimes W_{\mathrm{B}}^\dagger\right)\right]\right]. \tag{43}$$

In order to compute the expectation value $\langle\partial_\nu C\rangle_V$ we need to consider three different scenarios: (1) when only W_{A} is a 1-design; (2) when only W_{B} is a 1-design; and (3) when both W_{A} and W_{B} form 1-designs. We first consider the case when W_{A} is a 1-design. Since W_{A}, W_{B}, V_{R} and V_{L} are independent, we can compute the expectation value over the ansatz as $\langle\partial_\nu C\rangle_V = \langle\langle\partial_\nu C\rangle_{W_{\mathrm{A}}}\rangle_{V_{\mathrm{L}},W_{\mathrm{B}},V_{\mathrm{R}}}$. From (42) and the definition of a 1-design in (1), we can compute

$$\begin{aligned}\langle\partial_\nu C\rangle_{W_{\mathrm{A}}} &= -\frac{i}{2}\mathrm{Tr}\left[\left(\mathbb{1}_{\overline{w}}\otimes W_{\mathrm{B}}\right)V_{\mathrm{L}}\rho V_{\mathrm{L}}^\dagger\left(\mathbb{1}_{\overline{w}}\otimes W_{\mathrm{B}}^\dagger\right)\left[\mathbb{1}_{\overline{w}}\otimes\sigma_\nu,\int d\mu(W_{\mathrm{A}})\left(\mathbb{1}_{\overline{w}}\otimes W_{\mathrm{A}}\right)V_{\mathrm{R}}^\dagger O V_{\mathrm{R}}\left(\mathbb{1}_{\overline{w}}\otimes W_{\mathrm{A}}^\dagger\right)\right]\right]\\ &= -\frac{i}{2}\mathrm{Tr}\left[\left(\mathbb{1}_{\overline{w}}\otimes W_{\mathrm{B}}\right)V_{\mathrm{L}}\rho V_{\mathrm{L}}^\dagger\left(\mathbb{1}_{\overline{w}}\otimes W_{\mathrm{B}}^\dagger\right)\left[\mathbb{1}_{\overline{w}}\otimes\sigma_\nu,\frac{1}{2^m}\mathrm{Tr}_w[V_{\mathrm{R}}^\dagger O V_{\mathrm{R}}]\otimes\mathbb{1}_w\right]\right]\\ &= 0\,,\end{aligned} \tag{44}$$

where in the second equality we used Lemma 4.

On the other hand, if W_{B} is a 1-design, we can now employ (43) to get

$$\begin{aligned}\langle\partial_\nu C\rangle_{W_{\mathrm{B}}} &= -\frac{i}{2}\mathrm{Tr}\left[\left(\mathbb{1}_{\overline{w}}\otimes W_{\mathrm{A}}^\dagger\right)V_{\mathrm{R}}^\dagger O V_{\mathrm{R}}\left(\mathbb{1}_{\overline{w}}\otimes W_{\mathrm{A}}\right)\left[\mathbb{1}_{\overline{w}}\otimes\sigma_\nu,\int d\mu(W_{\mathrm{B}})\left(\mathbb{1}_{\overline{w}}\otimes W_{\mathrm{B}}\right)V_{\mathrm{L}}\rho V_{\mathrm{L}}^\dagger\left(\mathbb{1}_{\overline{w}}\otimes W_{\mathrm{B}}^\dagger\right)\right]\right]\\ &= -\frac{i}{2}\mathrm{Tr}\left[\left(\mathbb{1}_{\overline{w}}\otimes W_{\mathrm{A}}^\dagger\right)V_{\mathrm{R}}^\dagger O V_{\mathrm{R}}\left(\mathbb{1}_{\overline{w}}\otimes W_{\mathrm{A}}\right)\left[\mathbb{1}_{\overline{w}}\otimes\sigma_\nu,\frac{1}{2^m}\mathrm{Tr}_w[V_{\mathrm{L}}\rho V_{\mathrm{L}}^\dagger]\otimes\mathbb{1}_w\right]\right]\\ &= 0\,,\end{aligned} \tag{45}$$

which follows from the same argument used to derive (44). Finally, from Eqs. (44) and (45), we have $\langle\partial_\nu C\rangle_V = 0\ \ (\forall V_{\mathrm{L}}, V_{\mathrm{R}})$ if W_{A} and W_{B} are both 1-designs.

□

Supplementary Note 4: Variance of the cost function partial derivative

In this section, we derive the formula for the variance of the cost function gradient. Namely,

$$\langle(\partial_\nu C)^2\rangle_V = \frac{2^{m-1}\mathrm{Tr}[\sigma_\nu^2]}{(2^{2m}-1)^2}\sum_{\substack{\boldsymbol{pq}\\ \boldsymbol{p'q'}}}\left\langle\Delta\Omega_{\boldsymbol{pq}}^{\boldsymbol{p'q'}}\right\rangle_{V_{\mathrm{R}}}\left\langle\Delta\Psi_{\boldsymbol{pq}}^{\boldsymbol{p'q'}}\right\rangle_{V_{\mathrm{L}}}, \tag{46}$$

with

$$\Delta\Omega_{\boldsymbol{pq}}^{\boldsymbol{p'q'}} = \mathrm{Tr}[\Omega_{\boldsymbol{qp}}\Omega_{\boldsymbol{q'p'}}] - \frac{\mathrm{Tr}[\Omega_{\boldsymbol{qp}}]\mathrm{Tr}[\Omega_{\boldsymbol{q'p'}}]}{2^m}, \tag{47}$$

$$\Delta\Psi_{\boldsymbol{pq}}^{\boldsymbol{p'q'}} = \mathrm{Tr}[\Psi_{\boldsymbol{pq}}\Psi_{\boldsymbol{p'q'}}] - \frac{\mathrm{Tr}[\Psi_{\boldsymbol{pq}}]\mathrm{Tr}[\Psi_{\boldsymbol{p'q'}}]}{2^m}, \tag{48}$$

and where

$$\Omega_{\boldsymbol{qp}} = \mathrm{Tr}_{\overline{w}}\left[(|\boldsymbol{p}\rangle\langle\boldsymbol{q}|\otimes\mathbb{1}_w)V_{\mathrm{R}}^\dagger O V_{\mathrm{R}}\right], \tag{49}$$

$$\Psi_{\boldsymbol{pq}} = \mathrm{Tr}_{\overline{w}}\left[(|\boldsymbol{q}\rangle\langle\boldsymbol{p}|\otimes\mathbb{1}_w)V_{\mathrm{L}}\rho V_{\mathrm{L}}^\dagger\right]. \tag{50}$$

Proof. As shown in the previous section, $\langle\partial_\nu C\rangle_V = 0$ when either W_{B} or W_{A} of (39) are 1-designs. Hence, we can compute the variance of $\partial_\nu C$ as $\mathrm{Var}[\partial_\nu C] = \langle(\partial_\nu C)^2\rangle_V - \langle\partial_\nu C\rangle_V^2 = \langle(\partial_\nu C)^2\rangle_V$. From (43) and Lemma 5 we have

$$(\partial_\nu C)^2 = -\frac{1}{4}\sum_{\substack{\boldsymbol{p},\boldsymbol{q}\\ \boldsymbol{p'},\boldsymbol{q'}}}\mathrm{Tr}\left[W_{\mathrm{A}}\Omega_{\boldsymbol{qp}}W_{\mathrm{A}}^\dagger\Gamma_{\boldsymbol{pq}}\right]\mathrm{Tr}\left[W_{\mathrm{A}}\Omega_{\boldsymbol{q'p'}}W_{\mathrm{A}}^\dagger\Gamma_{\boldsymbol{p'q'}}\right], \tag{51}$$

with

$$\begin{aligned}\Gamma_{\boldsymbol{pq}} &= \mathrm{Tr}_{\overline{w}}\left[(|\boldsymbol{q}\rangle\langle\boldsymbol{p}|\otimes\mathbb{1}_w)\left[\mathbb{1}_{\overline{w}}\otimes\sigma_\nu,(\mathbb{1}_{\overline{w}}\otimes W_{\mathrm{B}})V_{\mathrm{L}}\rho V_{\mathrm{L}}^\dagger(\mathbb{1}_{\overline{w}}\otimes W_{\mathrm{B}}^\dagger)\right]\right]\\ &= \mathrm{Tr}_{\overline{w}}\left[[\mathbb{1}_{\overline{w}}\otimes\sigma_\nu,(\mathbb{1}_{\overline{w}}\otimes W_{\mathrm{B}})(|\boldsymbol{q}\rangle\langle\boldsymbol{p}|\otimes\mathbb{1}_w)V_{\mathrm{L}}\rho V_{\mathrm{L}}^\dagger(\mathbb{1}_{\overline{w}}\otimes W_{\mathrm{B}}^\dagger)]\right]\\ &= \left[\sigma_\nu, W_{\mathrm{B}}\mathrm{Tr}_{\overline{w}}[(|\boldsymbol{q}\rangle\langle\boldsymbol{p}|\otimes\mathbb{1}_w)V_{\mathrm{L}}\rho V_{\mathrm{L}}^\dagger]W_{\mathrm{B}}^\dagger\right]\\ &= \left[\sigma_\nu, W_{\mathrm{B}}\Psi_{\boldsymbol{pq}}W_{\mathrm{B}}^\dagger\right]\,. \end{aligned} \tag{52}$$

As previously mentioned, if W_{A}, W_{B}, V_{R} and V_{L} are independent, the expectation value of (51) can be computed as $\langle(\partial_\nu C)^2\rangle_V = \langle(\partial_\nu C)^2\rangle_{V_{\mathrm{L}},W_{\mathrm{B}},W_{\mathrm{A}},V_{\mathrm{R}}}$. In addition, if W_{A} is a 2-design, we get from Lemma 3

$$\begin{aligned}\langle(\partial_\nu C)^2\rangle_{W_{\mathrm{A}}} &= -\frac{1}{4}\sum_{\substack{\boldsymbol{p},\boldsymbol{q}\\ \boldsymbol{p}',\boldsymbol{q}'}}\int d\mu(W_{\mathrm{A}})\mathrm{Tr}\left[W_{\mathrm{A}}\Omega_{\boldsymbol{qp}}W_{\mathrm{A}}^\dagger\Gamma_{\boldsymbol{pq}}\right]\mathrm{Tr}\left[W_{\mathrm{A}}\Omega_{\boldsymbol{q}'\boldsymbol{p}'}W_{\mathrm{A}}^\dagger\Gamma_{\boldsymbol{p}'\boldsymbol{q}'}\right]\\ &= -\frac{1}{4}\sum_{\substack{\boldsymbol{p},\boldsymbol{q}\\ \boldsymbol{p}',\boldsymbol{q}'}}\left(\frac{1}{2^{2m}-1}\left(\mathrm{Tr}[\Omega_{\boldsymbol{qp}}]\mathrm{Tr}[\Gamma_{\boldsymbol{pq}}]\mathrm{Tr}[\Omega_{\boldsymbol{q}'\boldsymbol{p}'}]\mathrm{Tr}[\Gamma_{\boldsymbol{p}'\boldsymbol{q}'}]+\mathrm{Tr}[\Omega_{\boldsymbol{qp}}\Omega_{\boldsymbol{q}'\boldsymbol{p}'}]\mathrm{Tr}[\Gamma_{\boldsymbol{pq}}\Gamma_{\boldsymbol{p}'\boldsymbol{q}'}]\right)\right.\\ &\quad\left.-\frac{1}{2^m(2^{2m}-1)}\left(\mathrm{Tr}[\Omega_{\boldsymbol{qp}}\Omega_{\boldsymbol{q}'\boldsymbol{p}'}]\mathrm{Tr}[\Gamma_{\boldsymbol{pq}}]\mathrm{Tr}[\Gamma_{\boldsymbol{p}'\boldsymbol{q}'}]+\mathrm{Tr}[\Omega_{\boldsymbol{qp}}]\mathrm{Tr}[\Omega_{\boldsymbol{q}'\boldsymbol{p}'}]\mathrm{Tr}[\Gamma_{\boldsymbol{pq}}\Gamma_{\boldsymbol{p}'\boldsymbol{q}'}]\right)\right)\\ &= -\frac{1}{4(2^{2m}-1)}\sum_{\substack{\boldsymbol{p},\boldsymbol{q}\\ \boldsymbol{p}',\boldsymbol{q}'}}\left(\mathrm{Tr}[\Omega_{\boldsymbol{qp}}\Omega_{\boldsymbol{q}'\boldsymbol{p}'}]-\frac{1}{2^m}\mathrm{Tr}[\Omega_{\boldsymbol{qp}}]\mathrm{Tr}[\Omega_{\boldsymbol{q}'\boldsymbol{p}'}]\right)\mathrm{Tr}[\Gamma_{\boldsymbol{pq}}\Gamma_{\boldsymbol{p}'\boldsymbol{q}'}]\,,\end{aligned} \tag{53}$$

where in the third equality we used the fact that the trace of a commutator is zero: $\mathrm{Tr}[\Gamma_{\boldsymbol{pq}}] = 0$.

If W_{B} is also a 2-design, then from (53) we need to compute the following expectation value

$$\langle(\partial_\nu C)^2\rangle_{W_{\mathrm{B}},W_{\mathrm{A}}} = -\frac{1}{4(2^{2m}-1)}\sum_{\substack{\boldsymbol{p},\boldsymbol{q}\\ \boldsymbol{p}',\boldsymbol{q}'}}\left(\mathrm{Tr}[\Omega_{\boldsymbol{qp}}\Omega_{\boldsymbol{q}'\boldsymbol{p}'}]-\frac{1}{2^m}\mathrm{Tr}[\Omega_{\boldsymbol{qp}}]\mathrm{Tr}[\Omega_{\boldsymbol{q}'\boldsymbol{p}'}]\right)\int d\mu(W_{\mathrm{B}})\mathrm{Tr}[\Gamma_{\boldsymbol{pq}}\Gamma_{\boldsymbol{p}'\boldsymbol{q}'}]\,. \tag{54}$$

Let us first note that

$$\begin{aligned}\Gamma_{\boldsymbol{pq}}\Gamma_{\boldsymbol{p}'\boldsymbol{q}'} &= \left[\sigma_\nu, W_{\mathrm{B}}\Psi_{\boldsymbol{pq}}W_{\mathrm{B}}^\dagger\right]\left[\sigma_\nu, W_{\mathrm{B}}\Psi_{\boldsymbol{p}'\boldsymbol{q}'}W_{\mathrm{B}}^\dagger\right]\\ &= 2\left(\sigma_\nu W_{\mathrm{B}}\Psi_{\boldsymbol{pq}}W_{\mathrm{B}}^\dagger\sigma_\nu W_{\mathrm{B}}\Psi_{\boldsymbol{p}'\boldsymbol{q}'}W_{\mathrm{B}}^\dagger\right)-2\left(W_{\mathrm{B}}\sigma_\nu^2W_{\mathrm{B}}^\dagger\Psi_{\boldsymbol{pq}}\Psi_{\boldsymbol{p}'\boldsymbol{q}'}\right)\,.\end{aligned} \tag{55}$$

This result can be used along with Lemmas 1 and 2 to compute the integral in (55) as

$$\begin{aligned}\int d\mu(W_{\mathrm{B}})\mathrm{Tr}[\Gamma_{\boldsymbol{pq}}\Gamma_{\boldsymbol{p}'\boldsymbol{q}'}] =& 2\int d\mu(W_{\mathrm{B}})\mathrm{Tr}\left[\sigma_\nu W_{\mathrm{B}}\Psi_{\boldsymbol{pq}}W_{\mathrm{B}}^\dagger\sigma_\nu W_{\mathrm{B}}\Psi_{\boldsymbol{p}'\boldsymbol{q}'}W_{\mathrm{B}}^\dagger\right]\\ &-2\int d\mu(W_{\mathrm{B}})\mathrm{Tr}\left[W_{\mathrm{B}}\sigma_\nu^2W_{\mathrm{B}}^\dagger\Psi_{\boldsymbol{pq}}\Psi_{\boldsymbol{p}'\boldsymbol{q}'}\right]\\ =& -\frac{2^{m+1}}{2^{2m}-1}\mathrm{Tr}[\sigma_\nu^2]\left(\mathrm{Tr}[\Psi_{\boldsymbol{pq}}\Psi_{\boldsymbol{p}'\boldsymbol{q}'}]-\frac{1}{2^m}\mathrm{Tr}[\Psi_{\boldsymbol{pq}}]\mathrm{Tr}[\Psi_{\boldsymbol{p}'\boldsymbol{q}'}]\right)\,,\end{aligned} \tag{56}$$

where we used the fact that σ_ν is a Pauli operator, and hence its trace is equal to zero.

Then, combining Eqs. (54) and (56), we obtain

$$\langle(\partial_\nu C)^2\rangle_V = \frac{2^{m-1}\mathrm{Tr}[\sigma_\nu^2]}{(2^{2m}-1)^2}\sum_{\substack{\boldsymbol{pq}\\ \boldsymbol{p}'\boldsymbol{q}'}}\left\langle\Delta\Omega_{\boldsymbol{pq}}^{\boldsymbol{p}'\boldsymbol{q}'}\right\rangle_{V_{\mathrm{R}}}\left\langle\Delta\Psi_{\boldsymbol{pq}}^{\boldsymbol{p}'\boldsymbol{q}'}\right\rangle_{V_{\mathrm{L}}}\,. \tag{57}$$

□

Supplementary Note 5: Variance of the cost function partial derivative for a single layer of the Alternating Layered Ansatz

In this section we explicitly evaluate Eqs. (46)–(48) for the special case when $V(\boldsymbol{\theta})$ is composed of a single layer of the Alternating Layered Ansatz. This case is a generalization of the warm-up example of the main text, and constitutes the first step towards our main theorems. In particular, we remark that the tools employed here are the same as the ones used to derive our main result.

A. Variance of global cost function partial derivative

Let us first recall that the global cost function is $C_G = 1 - \text{Tr}\left[OV\rho V^\dagger\right]$, where $O = \bigotimes_{k=1}^{\xi} \widehat{O}_k$, and where $V(\boldsymbol{\theta})$ is given by a single layer of the Alternating Layered Ansatz, i.e., $V(\boldsymbol{\theta}) = \bigotimes_{k=1}^{\xi} W_{k1}(\boldsymbol{\theta}_k)$. Moreover, we recall that we assume without loss of generality that V_{R} contains the gates in $\mathcal{L}$ and all the blocks W_{k1} in the last (and in this case only) layer of $V(\boldsymbol{\theta})$. The latter means that here $V_{\text{L}} = \mathbb{1}$, and $V_{\text{R}} = \mathbb{1}_h \otimes \left(\bigotimes_{k\neq h} W_{k1}(\boldsymbol{\theta}_k)\right)$. In addition, for simplicity, we have defined $W_k := W_{k1}$. Here, ξ is the total number of blocks so that $n = \xi m$, and we assume that the angle θ_ν we want to train is in the h-th block W_h.

1. Expectation value over V_R

First, let us compute $\Omega_{\boldsymbol{qp}}$. From (49), we obtain

$$\Omega_{\boldsymbol{qp}} = \widehat{O}_h \prod_{k\neq h}^{\xi} \text{Tr}\left[W_k^\dagger \widehat{O}_k W_k |\boldsymbol{p}_k\rangle\langle \boldsymbol{q}_k|\right] . \tag{58}$$

Replacing this result in Eq. (47) and employing Lemma 3 $(\xi-1)$-times results in

$$\begin{aligned}\left\langle \Delta\Omega_{\boldsymbol{pq}}^{\boldsymbol{p'q'}}\right\rangle_{V_{\text{R}}} =& \frac{1}{(2^{2m}-1)^{\xi-1}}\left(\text{Tr}\left[\widehat{O}_h^2\right] - \frac{1}{2^m}\text{Tr}\left[\widehat{O}_h\right]^2\right)\\ &\times \prod_{k\neq h}^{\xi}\left(\delta_{(\boldsymbol{p},\boldsymbol{q})_{S_k}}\delta_{(\boldsymbol{p'},\boldsymbol{q'})_{S_k}}\left(\text{Tr}\left[\widehat{O}_k\right]^2 - \frac{1}{2^m}\text{Tr}\left[\widehat{O}_k^2\right]\right) + \delta_{(\boldsymbol{p'},\boldsymbol{q})_{S_k}}\delta_{(\boldsymbol{p},\boldsymbol{q'})_{S_k}}\left(\text{Tr}\left[\widehat{O}_k^2\right] - \frac{1}{2^m}\text{Tr}\left[\widehat{O}_k\right]^2\right)\right) .\end{aligned}$$

Now, let us consider $\widehat{O}_k$ $(k = 1, 2, \cdots, \xi)$ to be rank-1 projector, i.e. $\text{Tr}\left[\widehat{O}_k\right] = \text{Tr}\left[\widehat{O}_k^2\right] = \text{rank}\left[\widehat{O}_k\right] = 1$. Therefore, we can obtain

$$\left\langle \Delta\Omega_{\boldsymbol{pq}}^{\boldsymbol{p'q'}}\right\rangle_{V_{\text{R}}} = \frac{1}{(2^{2m}-1)^{\xi-1}}\left(1-\frac{1}{2^m}\right)^{\xi}\prod_{k\neq h}^{\xi}\left(\delta_{(\boldsymbol{p},\boldsymbol{q})_{S_k}}\delta_{(\boldsymbol{p'},\boldsymbol{q'})_{S_k}} + \delta_{(\boldsymbol{p'},\boldsymbol{q})_{S_k}}\delta_{(\boldsymbol{p},\boldsymbol{q'})_{S_k}}\right) . \tag{59}$$

2. Expectation value over V_L

Next, let us consider $\Psi_{\boldsymbol{pq}}$ in (50). Here, we can set $V_{\text{L}} = \mathbb{1}$, which leads to $\Psi_{\boldsymbol{pq}} = \text{Tr}_{\overline{h}}\left[\left(|\boldsymbol{p}\rangle\langle\boldsymbol{q}| \otimes \mathbb{1}_h\right)\rho\right]$. Note that any quantum state ρ can be always written as

$$\rho = \sum_\lambda p_\lambda |\psi_\lambda\rangle\langle\psi_\lambda| , \quad \text{with} \quad |\psi_\lambda\rangle = \sum_{\boldsymbol{\alpha}^\lambda} c_{\boldsymbol{\alpha}^\lambda} |\boldsymbol{\alpha}_1^\lambda\rangle \otimes \cdots \otimes |\boldsymbol{\alpha}_h^\lambda\rangle \otimes \cdots \otimes |\boldsymbol{\alpha}_\xi^\lambda\rangle , \tag{60}$$

where $\boldsymbol{\alpha} := \boldsymbol{\alpha_1}\cdots\boldsymbol{\alpha_\xi}$, and where $\boldsymbol{\alpha_i}$ are bitstrings of length m. Hence we find

$$\Psi_{\boldsymbol{pq}} = \sum_\lambda p_\lambda \sum_{\boldsymbol{\alpha}^\lambda, \boldsymbol{\alpha'}^\lambda} c_{\boldsymbol{\alpha}^\lambda} c^*_{\boldsymbol{\alpha'}^\lambda}\left(\prod_{k\neq h}^{\xi} \delta_{(\boldsymbol{q},\boldsymbol{\alpha}^\lambda)_k}\delta_{(\boldsymbol{p},\boldsymbol{\alpha'}^\lambda)_{S_k}}\right)|\boldsymbol{\alpha}_h^\lambda\rangle\langle\boldsymbol{\alpha'}_h^\lambda| . \tag{61}$$

Then, since $V_{\rm L}=\mathbb{1}$, we have $\left\langle \Delta\Psi_{\boldsymbol{pq}}^{\boldsymbol{p'q'}}\right\rangle_{V_{\rm L}} = \Delta\Psi_{\boldsymbol{pq}}^{\boldsymbol{p'q'}}$, and we can use (61) to get

$$\begin{aligned}\Delta\Psi_{\boldsymbol{pq}}^{\boldsymbol{p'q'}} =& \sum_{\lambda,\lambda'} p_\lambda p_{\lambda'} \sum_{\substack{\boldsymbol{\alpha}^\lambda,\boldsymbol{\alpha'}^\lambda\\ \boldsymbol{\beta}^{\lambda'},\boldsymbol{\beta'}^{\lambda'}}} c_{\boldsymbol{\alpha}^\lambda} c^*_{\boldsymbol{\alpha'}^\lambda} c_{\boldsymbol{\beta}^{\lambda'}} c^*_{\boldsymbol{\beta'}^{\lambda'}} \left(\prod_{k\neq h}^{\xi} \delta_{(\boldsymbol{q},\boldsymbol{\alpha}^\lambda)_{S_k}} \delta_{(\boldsymbol{p},\boldsymbol{\alpha'}^\lambda)_{S_k}} \delta_{(\boldsymbol{q'},\boldsymbol{\beta}^{\lambda'})_{S_k}} \delta_{(\boldsymbol{p'},\boldsymbol{\beta'}^{\lambda'})_{S_k}} \right)\\ &\times \left(\delta_{(\boldsymbol{\alpha'}^\lambda,\boldsymbol{\beta}^{\lambda'})_{S_h}} \delta_{(\boldsymbol{\alpha}^\lambda,\boldsymbol{\beta'}^{\lambda'})_{S_h}} - \frac{1}{2^m} \delta_{(\boldsymbol{\alpha}^\lambda,\boldsymbol{\alpha'}^\lambda)_{S_h}} \delta_{(\boldsymbol{\beta}^{\lambda'},\boldsymbol{\beta'}^{\lambda'})_{S_h}} \right) .\end{aligned} \tag{62}$$

Finally, from (59), (62), and the fact that $\mathrm{Tr}\left[\sigma_\nu^2\right]=2^m$, we obtain

$$\begin{aligned}\mathrm{Var}\left[\partial_\nu C_G\right] =& \frac{2^{2m-1}}{(2^{2m}-1)^2(2^{2m}-1)^{\xi-1}} \left(1-\frac{1}{2^m}\right)^{\xi} \sum_{\lambda,\lambda'} p_\lambda p_{\lambda'} \sum_{\substack{\boldsymbol{\alpha}^\lambda,\boldsymbol{\alpha'}^\lambda\\ \boldsymbol{\beta}^{\lambda'},\boldsymbol{\beta'}^{\lambda'}}} c_{\boldsymbol{\alpha}^\lambda} c^*_{\boldsymbol{\alpha'}^\lambda} c_{\boldsymbol{\beta}^{\lambda'}} c^*_{\boldsymbol{\beta'}^{\lambda'}}\\ &\times \left(\delta_{(\boldsymbol{\alpha'}^\lambda,\boldsymbol{\beta}^{\lambda'})_{S_h}} \delta_{(\boldsymbol{\alpha}^\lambda,\boldsymbol{\beta'}^{\lambda'})_{S_h}} - \frac{1}{2^m} \delta_{(\boldsymbol{\alpha}^\lambda,\boldsymbol{\alpha'}^\lambda)_{S_h}} \delta_{(\boldsymbol{\beta}^{\lambda'},\boldsymbol{\beta'}^{\lambda'})_{S_h}} \right) \prod_{k\neq h} \left(\delta_{(\boldsymbol{\alpha}^\lambda,\boldsymbol{\alpha'}^\lambda)_{S_k}} \delta_{(\boldsymbol{\beta}^{\lambda'},\boldsymbol{\beta'}^{\lambda'})_{S_k}} + \delta_{(\boldsymbol{\alpha}^\lambda,\boldsymbol{\beta'}^{\lambda'})_{S_k}} \delta_{(\boldsymbol{\alpha'}^\lambda,\boldsymbol{\beta}^{\lambda'})_{S_k}} \right) ,\end{aligned}$$

where we used the fact that

$$\begin{aligned}&\sum_{\substack{\boldsymbol{pq}\\ \boldsymbol{p'q'}}} \left(\prod_{k\neq h}^{\xi} \left(\delta_{(\boldsymbol{p},\boldsymbol{q})_{S_k}} \delta_{(\boldsymbol{p'},\boldsymbol{q'})_{S_k}} + \delta_{(\boldsymbol{p'},\boldsymbol{q})_{S_k}} \delta_{(\boldsymbol{p},\boldsymbol{q'})_{S_k}} \right) \delta_{(\boldsymbol{q},\boldsymbol{\alpha}^\lambda)_{S_k}} \delta_{(\boldsymbol{p},\boldsymbol{\alpha'}^\lambda)_{S_k}} \delta_{(\boldsymbol{q'},\boldsymbol{\beta}^{\lambda'})_{S_k}} \delta_{(\boldsymbol{p'},\boldsymbol{\beta'}^{\lambda'})_{S_k}} \right)\\ &\quad = \prod_{k\neq h} \left(\delta_{(\boldsymbol{\alpha}^\lambda,\boldsymbol{\alpha'}^\lambda)_{S_k}} \delta_{(\boldsymbol{\beta}^{\lambda'},\boldsymbol{\beta'}^{\lambda'})_{S_k}} + \delta_{(\boldsymbol{\alpha}^\lambda,\boldsymbol{\beta'}^{\lambda'})_{S_k}} \delta_{(\boldsymbol{\alpha'}^\lambda,\boldsymbol{\beta}^{\lambda'})_{S_k}} \right) .\end{aligned}$$

Let us define

$$\begin{aligned}J :=& \sum_{\lambda,\lambda'} p_\lambda p_{\lambda'} \sum_{\substack{\boldsymbol{\alpha}^\lambda,\boldsymbol{\alpha'}^\lambda\\ \boldsymbol{\beta}^{\lambda'},\boldsymbol{\beta'}^{\lambda'}}} c_{\boldsymbol{\alpha}^\lambda} c^*_{\boldsymbol{\alpha'}^\lambda} c_{\boldsymbol{\beta}^{\lambda'}} c^*_{\boldsymbol{\beta'}^{\lambda'}}\\ &\times \left(\delta_{(\boldsymbol{\alpha'}^\lambda,\boldsymbol{\beta}^{\lambda'})_{S_h}} \delta_{(\boldsymbol{\alpha}^\lambda,\boldsymbol{\beta'}^{\lambda'})_{S_h}} - \frac{1}{2^m} \delta_{(\boldsymbol{\alpha}^\lambda,\boldsymbol{\alpha'}^\lambda)_{S_h}} \delta_{(\boldsymbol{\beta}^{\lambda'},\boldsymbol{\beta'}^{\lambda'})_{S_h}} \right) \prod_{k\neq h} \left(\delta_{(\boldsymbol{\alpha}^\lambda,\boldsymbol{\alpha'}^\lambda)_{S_k}} \delta_{(\boldsymbol{\beta}^{\lambda'},\boldsymbol{\beta'}^{\lambda'})_{S_k}} + \delta_{(\boldsymbol{\alpha}^\lambda,\boldsymbol{\beta'}^{\lambda'})_{S_k}} \delta_{(\boldsymbol{\alpha'}^\lambda,\boldsymbol{\beta}^{\lambda'})_{S_k}} \right) ,\end{aligned}$$

which, has the form of $J = 1-\frac{1}{2^m}+\sum_{l=1}^{2^{\xi-1}-1}\left(\mathcal{A}_{\rm L}-\frac{1}{2^m}\mathcal{B}_{\rm L}\right)$, where $\mathcal{A}_{\rm L}$, $\mathcal{B}_{\rm L}$ are the purities of reduced states of ρ. The latter can be understood in the following way. Let us define the set $\mathcal{S}=\{S_1,S_2,\cdots,S_\xi\}$, whose elements represents each block. Then, suppose that we want to consider the partial trace of ρ over several subsystems $\mathcal{H}_{\overline{\boldsymbol{K}}}$, where $\overline{\boldsymbol{K}}\subset\mathcal{S}$. The reduced state lives in the composite Hilbert space $\mathcal{H}_{\boldsymbol{K}}$, where $\boldsymbol{K}=\mathcal{S}\setminus\overline{\boldsymbol{K}}$, and we can write $\boldsymbol{\alpha}:=\boldsymbol{\alpha}_{\boldsymbol{K}}\cdot\boldsymbol{\alpha}_{\overline{\boldsymbol{K}}}$. Let us define the reduced state as $\rho_{\boldsymbol{K}}:=\mathrm{Tr}_{\overline{\boldsymbol{K}}}[\rho]$, and its purity can be explicitly written as

$$\mathrm{Tr}\left[\rho_{\boldsymbol{K}}^2\right] = \sum_{\lambda,\lambda'} p_\lambda p_{\lambda'} \sum_{\boldsymbol{\alpha}^\lambda,\boldsymbol{\beta}^{\lambda'}} c_{\boldsymbol{\alpha}^\lambda} c^*_{\boldsymbol{\alpha}^\lambda_{\overline{\boldsymbol{K}}}\cdot\boldsymbol{\beta}^{\lambda'}_{\boldsymbol{K}}} c_{\boldsymbol{\beta}^{\lambda'}} c^*_{\boldsymbol{\alpha}^\lambda_{\boldsymbol{K}}\cdot\boldsymbol{\beta}^{\lambda'}_{\overline{\boldsymbol{K}}}} ,$$

which appears in the expression of J. Since $\mathrm{Tr}\left[\rho_{\boldsymbol{K}}^2\right]\leqslant 1$, we have $J<2^{\xi-1}$; therefor, we can write

$$\mathrm{Var}\left[\partial_\nu C_G\right] < \frac{2^{2m-2}}{(2^{2m}-1)2^{2m\xi-\xi}(1+2^{-m})^{\xi}} < \frac{1}{2^{\left(2-\frac{1}{m}\right)n}} \quad (\forall m\in\mathbb{N}) , \tag{63}$$

where in the last inequality we used the fact that $n=m\xi$. Equation (63) shows that for the single layer of the alternating layered ansatz the global cost functions presents a barren plateau.

B. Variance of the local cost function partial derivative

Here we consider the case when $V(\boldsymbol{\theta})$ is given by a single layer of the Alternating Layered Ansatz, and when the local cost function is

$$C_{\rm L} = 1-\frac{1}{n}\sum_{j=1}^{n}\mathrm{Tr}\left[\left(|0\rangle\langle 0|_j\otimes\mathbb{1}_{\overline{j}}\right)V\rho V^\dagger\right] .$$

with $O_{\rm L} = \frac{1}{n}\sum_{j=1}^{n} |0\rangle\langle 0|_j \otimes \mathbb{1}_{\overline{j}}$, and where j denotes the j-th qubit. Moreover, let us assume that we are training a parameter θ_ν in h-th block. This case is simpler than the one previously considered for the global cost function as the gradient is non vanishing only when we measure a qubit j in S_h. We can then redefine $O_{\rm L}^h : \mathcal{H}_h \to \mathcal{H}_h$ as $\widehat{O}_{\rm L}^h = \frac{1}{n}\sum_{j=1}^{m} |0\rangle\langle 0|_j \otimes \mathbb{1}_{\overline{j}}$, where each j is such that $j \in S_h$. Then, from (57) we have

$$\mathrm{Tr}\left[\widehat{O}_{\rm L}^h\right] = \frac{m2^{m-1}}{n}\,, \quad \mathrm{Tr}\left[\left(\widehat{O}_{\rm L}^h\right)^2\right] = \frac{m(m+1)2^{m-2}}{n^2}\,. \tag{64}$$

From (49), $\Omega_{\boldsymbol{qp}}$ can be written as $\Omega_{\boldsymbol{qp}} = \delta_{\boldsymbol{pq}} W_h O_{\rm L}^h W_h^\dagger$, which leads to

$$\Delta\Omega_{\boldsymbol{pq}}^{\boldsymbol{p'q'}} = \frac{m2^{m-2}}{n^2}\delta_{\boldsymbol{pq}}\delta_{\boldsymbol{p'q'}}\,. \tag{65}$$

Next, from (50) we have $\Psi_{\boldsymbol{pq}} = \mathrm{Tr}_{\overline{h}}\left[\left(|\boldsymbol{q}\rangle\langle\boldsymbol{p}| \otimes \mathbb{1}_h\right)\rho\right]$, and it is straightforward to see that

$$\sum_{\boldsymbol{pq}} \delta_{\boldsymbol{pq}}\Psi_{\boldsymbol{pq}} = \sum_{\boldsymbol{pq}} \delta_{\boldsymbol{pq}} \mathrm{Tr}_{\overline{h}}\left[\left(|\boldsymbol{q}\rangle\langle\boldsymbol{p}| \otimes \mathbb{1}_h\right)\rho\right] = \rho_h\,, \tag{66}$$

where we defined $\rho_h := \mathrm{Tr}_{\overline{h}}[\rho]$. Then, from (57), and by using $\left\langle \Delta\Psi_{\boldsymbol{pq}}^{\boldsymbol{p'q'}}\right\rangle_{V_{\rm L}} = \Delta\Psi_{\boldsymbol{pq}}^{\boldsymbol{p'q'}}$, and $\mathrm{Tr}[\sigma_\nu^2] = 2^m$, we can write

$$\mathrm{Var}\left[\partial_\nu C_{\rm L}\right] = \frac{m2^{3(m-1)}}{n^2(2^{2m}-1)^2}\left(\mathrm{Tr}[\rho_h^2] - \frac{1}{2^m}\right) = \frac{m\cdot 2^{3(m-1)}}{n^2(2^{2m}-1)^2} D_{HS}\left(\rho_h, \frac{\mathbb{1}}{2^m}\right)\,,$$

where $D_{HS}\left(\rho_h, \mathbb{1}/2^m\right) = \mathrm{Tr}[(\rho_h - \mathbb{1}/2^m)^2]$ is the Hilbert-Schmidt distance between ρ_h and $\mathbb{1}/2^m$. If $D_{HS}\left(\rho_h, \mathbb{1}/2^m\right) \in \Omega\left(1/\operatorname{poly}(n)\right)$, we have that the variance of the cost function partial derivative is polinomially vanishing with n as

$$\mathrm{Var}[\partial_\nu C_{\rm L}] \in \Omega\left(\frac{1}{\operatorname{poly}(n)}\right)\,, \tag{67}$$

and hence in this case $C_{\rm L}$ presents no barren plateau.

Supplementary Note 6: Proof of Theorem 2

First, let us recall that we are considering m-local cost functions where each operator O_i acts nontrivially on m qubits $O_i = \mathbb{1}_{\overline{m}} \otimes \widehat{O}_i$ (here $\mathbb{1}_{\overline{m}}$ indicates the identity on all but m qubits), and where $\widehat{O}_i$ can be expressed as $\widehat{O}_i = \widehat{O}_i^{\mu_i} \otimes \widehat{O}_i^{\mu_i'}$. Hence, we have

$$O = c_0\mathbb{1} + \sum_{i=1}^{N} c_i \widehat{O}_i^{\mu_i} \otimes \widehat{O}_i^{\mu_i'}\,, \tag{68}$$

where $\widehat{O}_i^{\mu_i}$ are operators acting on $m/2$ qubits which can be written as tensor product of Pauli operators. Here we recall that we have defined S_k as the m-qubit subsystem on which W_{kL} acts and let $\mathcal{S} = \{S_k\}$ be the set of all such subsystems. As detailed in the main text the summation in Eq. (68) includes two possible cases: First, when $\widehat{O}_i^{\mu_i}$ ($\widehat{O}_i^{\mu_i'}$) acts on the first (last) $m/2$ qubits of a given S_k, and second, when $\widehat{O}_i^{\mu_i}$ ($\widehat{O}_i^{\mu_i'}$) acts on the last (first) $m/2$ qubits of a given S_k (S_{k+1}). This type of cost function includes any ultralocal (i.e., where the O_i are one-body) cost function, and also VQE Hamiltonians with up to $m/2$ neighbor interactions.

Here we provide a proof of Theorem 2 in the main text, which we now reiterate for convenience.

Theorem 2. Consider a trainable parameter θ^ν in a block W of the ansatz in Fig. 3 of the main text. Let $\mathrm{Var}[\partial_\nu C]$ be the variance of the partial derivative of an m-local cost function C (with O given by (68)) with respect to θ^ν. If $W_{\rm A}$, $W_{\rm B}$ of (39), and each block in $V(\boldsymbol{\theta})$ form a local 2-design, then $\mathrm{Var}[\partial_\nu C]$ is lower bounded by

$$G_n(L,l) \leqslant \mathrm{Var}[\partial_\nu C]\,, \tag{69}$$

with

$$G_n(L,l) = \frac{2^{m(l+1)-1}}{(2^{2m}-1)^2(2^m+1)^{L+l}} \sum_{i\in i_{\mathcal{L}}} \sum_{\substack{(k,k')\in k_{\mathcal{L}_{\rm B}} \\ k'\geqslant k}} c_i^2 \epsilon(\rho_{k,k'})\epsilon(\widehat{O}_i)\,, \tag{70}$$

where $i_{\mathcal{L}}$ is the set of i indices whose associated operators $\widehat{O}_i$ act on qubits in the forward light-cone $\mathcal{L}$ of W, and $k_{\mathcal{L}_{\rm B}}$ is the set of k indices whose associated subsystems S_k are in the backward light-cone $\mathcal{L}_{\rm B}$ of W. Here we defined the function $\epsilon(M) = D_{\rm HS}\left(M, {\rm Tr}(M)\mathbb{1}/d_M\right)$ where $D_{\rm HS}$ is the Hilbert-Schmidt distance and d_M is the dimension of the matrix M. In addition, $\rho_{k,k'}$ is partial trace of the input state ρ down to the subsystems $S_k S_{k+1} ... S_{k'}$.

Proof. Let us first consider the case when the operators O_i are of the form (68) and act non trivially in a given subsystem of $\mathcal{S}$. We can expand

$$\text{Var}[\partial_\nu C] = \frac{2^{m-1}{\rm Tr}[\sigma_\nu^2]}{(2^{2m}-1)^2} \sum_{i,j} \sum_{\substack{\boldsymbol{pq}\\ \boldsymbol{p'q'}}} c_i c_j \left\langle {\rm Tr}[\Omega^i_{\boldsymbol{qp}} \Omega^j_{\boldsymbol{q'p'}}] - \frac{{\rm Tr}[\Omega^i_{\boldsymbol{qp}}]{\rm Tr}[\Omega^j_{\boldsymbol{q'p'}}]}{2^m} \right\rangle_{V_{\rm R}} \left\langle \Delta\Psi^{\boldsymbol{p'q'}}_{\boldsymbol{pq}} \right\rangle_{V_{\rm L}} , \tag{71}$$

where we defined

$$\Omega^i_{\boldsymbol{qp}} = {\rm Tr}_{\overline{w}}\left[(|\boldsymbol{p}\rangle\langle \boldsymbol{q}| \otimes \mathbb{1}_w) V_{\rm R}^\dagger O_i V_{\rm R} \right] . \tag{72}$$

1. *Expectation value over V_R*

We recall here that we assume without loss of generality that $V_{\rm R}$ contains the gates in $\mathcal{L}$ and all the blocks W_{kL} in the last layer of $V(\boldsymbol{\theta})$. Hence, we can express

$$V_{\rm R} = V_{\overline{\mathcal{L}}} \otimes V_{\mathcal{L}} , \tag{73}$$

where $V_{\mathcal{L}}$ contains all the blocks in the forward light-cone $\mathcal{L}$ of W, and where $V_{\overline{\mathcal{L}}}$ consists of all the remaining blocks in the last layer of the ansatz. When analyzing the operators $\Omega^i_{\boldsymbol{pq}}$ we have to consider two cases: 1) when O_i only acts non-trivially on qubits in $S_{\overline{\mathcal{L}}}$, and 2) when $\widehat{O}_i$ acts non-trivially on qubits in $S_{\mathcal{L}}$. If O_i only acts non-trivially on qubits in $S_{\overline{\mathcal{L}}}$, it is straightforward to show from (72) that $\Omega^i_{\boldsymbol{pq}} \propto \mathbb{1}_w$. Hence, in this case, we find

$${\rm Tr}[\Omega^i_{\boldsymbol{qp}} \Omega^j_{\boldsymbol{q'p'}}] - \frac{{\rm Tr}[\Omega^i_{\boldsymbol{qp}}]{\rm Tr}[\Omega^j_{\boldsymbol{q'p'}}]}{2^m} = 0 . \tag{74}$$

Let us then define $i_{\mathcal{L}}$ as the set of i indices whose associated operators O_i act on qubits in the forward light-cone $\mathcal{L}$ of W. In what follows we assume that the indexes $i, j \in i_{\mathcal{L}}$, i.e., we assume that O_i and O_j act non-trivially on qubit in $S_{\mathcal{L}}$. The latter leads to

$$\left\langle {\rm Tr}[\Omega^i_{\boldsymbol{qp}} \Omega^j_{\boldsymbol{q'p'}}] - \frac{{\rm Tr}[\Omega^i_{\boldsymbol{qp}}]{\rm Tr}[\Omega^j_{\boldsymbol{q'p'}}]}{2^m} \right\rangle_{V_{\rm R}} = \delta_{(\boldsymbol{p},\boldsymbol{q})_{S_{\overline{\mathcal{L}}}}} \delta_{(\boldsymbol{p'},\boldsymbol{q'})_{S_{\overline{\mathcal{L}}}}} \left\langle {\rm Tr}[\Omega^i_{\boldsymbol{qp}} \Omega^j_{\boldsymbol{q'p'}}] - \frac{{\rm Tr}[\Omega^i_{\boldsymbol{qp}}]{\rm Tr}[\Omega^j_{\boldsymbol{q'p'}}]}{2^m} \right\rangle_{V_{\mathcal{L}}} . \tag{75}$$

Here the delta functions arise from (72) by noting that $V_{\rm R}^\dagger O_i V_{\rm R} = V_{\mathcal{L}}^\dagger O_i V_{\mathcal{L}}$.

In order to explicitly evaluate the expectation value in (75) we use the fact that each block in the layered ansatz is a 2-design, and hence one can algorithmically integrate over each block using the Weingarten calculus. Specifically, as discussed in the main text we employ the tensor network representation of ${\rm Tr}[\Omega^i_{\boldsymbol{qp}} \Omega^i_{\boldsymbol{q'p'}}]$ and ${\rm Tr}[\Omega^i_{\boldsymbol{qp}}]{\rm Tr}[\Omega^j_{\boldsymbol{q'p'}}]$, and we use the *Random Tensor Network Integrator* (RTNI) package of Ref. [5], which allows for the computation of averages of tensor networks containing multiple Haar-distributed random unitary matrices and deterministic symbolic tensors.

Using this procedure, $\langle \ldots \rangle_{V_{\mathcal{L}}}$ can be computed by integrating over each block inside of $V_{\mathcal{L}}$ over the unitary group with respect to the Haar measure. After each integration the result of the average is a sum of up to four new tensor according to (3), and Lemmas 2 and 3. After all the blocks in $V_{\mathcal{L}}$ have been integrated, the final result can be expressed as

$$\left\langle {\rm Tr}[\Omega^i_{\boldsymbol{qp}} \Omega^j_{\boldsymbol{q'p'}}] - \frac{{\rm Tr}[\Omega^i_{\boldsymbol{qp}}]{\rm Tr}[\Omega^j_{\boldsymbol{q'p'}}]}{2^m} \right\rangle_{V_{\mathcal{L}}} = \sum_\tau t^{ij}_\tau \delta_{(\boldsymbol{p},\boldsymbol{q})_{S_{\overline{\tau}}}} \delta_{(\boldsymbol{p'},\boldsymbol{q'})_{S_{\overline{\tau}}}} \delta_{(\boldsymbol{p},\boldsymbol{q'})_{S_\tau}} \delta_{(\boldsymbol{p'},\boldsymbol{q})_{S_\tau}} \Delta O^{ij}_\tau , \tag{76}$$

where $t_\tau \in \mathbb{R}$, $S_\tau \cup S_{\overline{\tau}} = S_{\mathcal{L}} \cap S_{\overline{w}}$ (with $S_\tau \neq \emptyset$), and where we have defined

$$\Delta O^{ij}_\tau = {\rm Tr}_{x_\tau y_\tau}\left[{\rm Tr}_{z_\tau}[O_i]\, {\rm Tr}_{z_\tau}[O_j] \right] - \frac{{\rm Tr}_{x_\tau}\left[{\rm Tr}_{y_\tau z_\tau}[O_i]\, {\rm Tr}_{y_\tau z_\tau}[O_j] \right]}{2^m} . \tag{77}$$

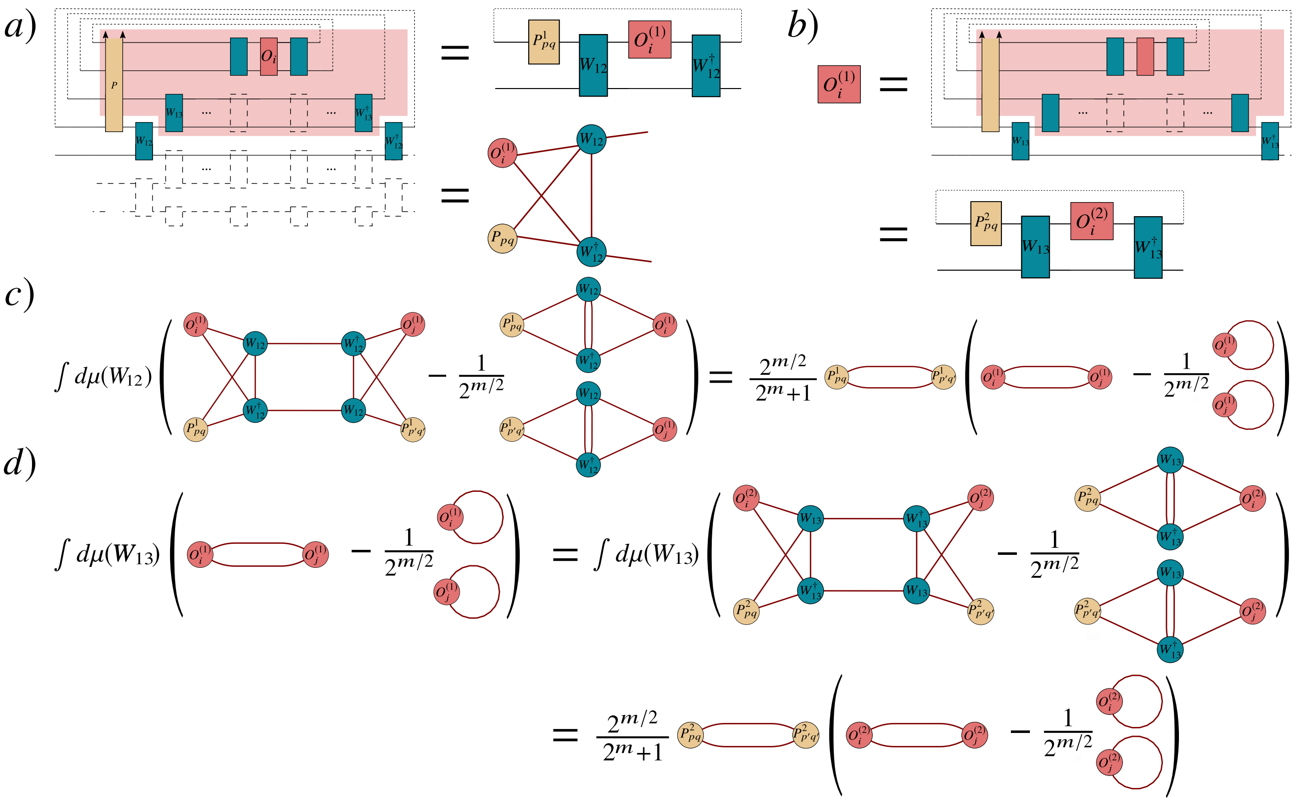

Supplementary Figure 1. Schematic representation of the method employed to derive Eq. (76). Here we consider the case when O_i and O_j act nontrivially only on the topmost m qubits in $V_{\mathcal{L}}$. (a) Tensor-network representation of the term $\Omega^i_{\boldsymbol{qp}}$. All but $L-l$ blocks in $V_{\mathcal{L}}$ simplify to identity when computing $V^\dagger_{\mathcal{L}} O_i V_{\mathcal{L}}$. One can always define operators $O^{(1)}_i$, and $O^{(1)}_j$ such that Eq. (78) holds. Here W_{12} is the topmost block in the first layer of $V_{\mathcal{L}}$, and we define $P^1_{\boldsymbol{pq}}$ as a projector operator acing on $m/2$ qubits. (b) Tensor-network representation of the operator $O^{(1)}_i$. Note that from $O^{(1)}_i$ we can also define an operator $O^{(2)}_i$ which will be of the same form as $O^{(1)}_i$. (c) By means of the Weingarten calculus we can integrate over W_{12} to compute the expectation value of Eq. (78). The result is a single tensor. This result can be obtained by employing Eq. (23) of Lemma 6. (d) In order the integrate the next block we use the operators $O^{(2)}_i$ to express the tensor networks in a form that coincides with panel (c). We can then repeat this procedure $L-l$ times in order to integrate over all blocks and compute the expectation value in (75). The final result is of the form of Eq. (79).

Here we use the notation Tr_{x_τ} to indicate the trace over the Hilbert space associated with subsystem S_{x_τ}. We also define $\mathcal{S}^{\mathcal{L}} = \{S_k : S_k \subset S_{\mathcal{L}}\}$ as the set of subspaces S_k which belong to the light-cone $\mathcal{L}$, so that we have $S_{y_\tau} \in \mathcal{S}^{\mathcal{L}}$, $S_{x_\tau} \cup S_{y_\tau} \cup S_{z_\tau} = S_{\mathcal{L}}$, and $S_{x_\tau}, S_{z_\tau} \in \mathcal{P}(\mathcal{S}^{\mathcal{L}})$, with $\mathcal{P}(\mathcal{S}^{\mathcal{L}}) = \{\emptyset, S_1, \ldots, S_\xi, S_1 \cup S_2 \ldots\}$ the power set of $\mathcal{S}^{\mathcal{L}}$. If one chooses $S_{z_\tau} = \emptyset$ (or $S_{x_\tau} = \emptyset$), with associated Hilbert space $H_0 = \{\mathbf{0}\}$, we define $\text{Tr}_\emptyset [O] := O$.

Equation (76) can be derived by iteratively applying Lemma 6 each time a block in $V_{\mathcal{L}}$ is integrated in (75), and noting that at each integration the resulting tensor can always be written in a way such that Lemma 6 can be applied again. As an example, let us consider the case when O_i and O_j act nontrivially only on the topmost m qubits in $V_{\mathcal{L}}$. As shown in Sup. Fig. 1(a), all but $L-l$ blocks in $V_{\mathcal{L}}$ simplify to identity when computing $V^\dagger_{\mathcal{L}} O_i V_{\mathcal{L}}$. Then, let us denote by W_{12} the topmost gate in the first layer in $V_{\mathcal{L}}$, and let $\widetilde{S}_1$, $\widetilde{S}_2$ be the sets of $m/2$ adjacent qubits (with associated Hilbert spaces $\widetilde{\mathcal{H}}_1$, $\widetilde{\mathcal{H}}_2$) such that W_{12} acts on $\widetilde{\mathcal{H}}_1 \otimes \widetilde{\mathcal{H}}_2$, and such that $\widetilde{S}_2 \subset S_w$. One can always write

$$\left\langle \text{Tr}[\Omega^i_{\boldsymbol{qp}} \Omega^j_{\boldsymbol{q'p'}}] - \frac{\text{Tr}[\Omega^i_{\boldsymbol{qp}}]\text{Tr}[\Omega^j_{\boldsymbol{q'p'}}]}{2^m} \right\rangle_{V_{\mathcal{L}}} \propto \left\langle \text{Tr}_{\widetilde{\mathcal{H}}_2}\left[\text{Tr}_{\widetilde{\mathcal{H}}_1}[\Omega_1] \text{Tr}_{\widetilde{\mathcal{H}}_1}[\Omega'_1] \right] - \frac{\text{Tr}[\Omega_1]\text{Tr}[\Omega'_1]}{2^m} \right\rangle_{V_{\mathcal{L}}}, \tag{78}$$

with $\Omega_1 = W_{12} O^{(1)}_i W^\dagger_{12} (|\boldsymbol{p}\rangle\langle \boldsymbol{q}|_{\widetilde{S}_1} \otimes \mathbb{1}_{\widetilde{S}_2})$, $\Omega'_1 = W_{12} O^{(1)}_j W^\dagger_{12} (|\boldsymbol{p}'\rangle\langle \boldsymbol{q}'|_{\widetilde{S}_1} \otimes \mathbb{1}_{\widetilde{S}_2})$, and where $O^{(1)}_i$, and $O^{(1)}_j$ are defined according to Sup. Fig. 1(a). The proportionallity factor in Eq. (78) is given by delta functions over $\boldsymbol{p}$, and $\boldsymbol{q}$. Moreover, the right-hand side of (78) is exactly of the form (16), and hence by applying Lemma 6 (or more specifically, Eq. (23)) one finds the result of Sup. Fig. 1(c). As schematically depicted in Sup. Fig. 1(b), one can then define operators $O^{(2)}_i$

and repeat this calculation $L-l$ times as in Sup. Fig. 1(d). One finally finds

$$\left\langle \text{Tr}[\Omega^i_{\boldsymbol{qp}}\Omega^j_{\boldsymbol{q}'\boldsymbol{p}'}] - \frac{\text{Tr}[\Omega^i_{\boldsymbol{qp}}]\text{Tr}[\Omega^j_{\boldsymbol{q}'\boldsymbol{p}'}]}{2^m} \right\rangle_{V_{\mathcal{L}}} \propto \frac{2^{m(L-l)/2}}{(2^m+1)^{L-l}} \left(\text{Tr}\left[O_i O_j\right] - \frac{\text{Tr}\left[O_i\right]\text{Tr}\left[O_j\right]}{2^m} \right), \tag{79}$$

where once again the proportionally factor is given by delta functions over $\boldsymbol{p}$ and $\boldsymbol{q}$. For more general operators O_i and O_j, each time a block is integrated, one can always rewrite the resulting non-trivial terms in the form of either Eq. (16), or Eq. (20). Remarkably, this means that the final result can always be expressed in terms of contractions of the form (77), and hence Eq. (76) holds.

As previously mentioned, Eq. (76) is valid for arbitrary operators O_i and O_j. However, from Lemma 7 we know that if O_i and O_j have no overlapping support, then $\Delta O^{ij}_\tau = 0$, for all τ. Hence we only have to consider the cases when $i=j$. Moreover, if $\widehat{O}_i$ act non trivially in a given subsystem of $\mathcal{S}$ the summation in Eq. (76) simplifies and we find

$$\left\langle \text{Tr}[\Omega^i_{\boldsymbol{qp}}\Omega^j_{\boldsymbol{q}'\boldsymbol{p}'}] - \frac{\text{Tr}[\Omega^i_{\boldsymbol{qp}}]\text{Tr}[\Omega^j_{\boldsymbol{q}'\boldsymbol{p}'}]}{2^m} \right\rangle_{V_{\mathcal{L}}} = \epsilon(\widehat{O}_i)\sum_\tau \hat{t}^{ii}_\tau \delta_{(\boldsymbol{p},\boldsymbol{q})_{S_{\overline{\tau}}}}\delta_{(\boldsymbol{p}',\boldsymbol{q}')_{S_{\overline{\tau}}}}\delta_{(\boldsymbol{p},\boldsymbol{q}')_{S_\tau}}\delta_{(\boldsymbol{p}',\boldsymbol{q})_{S_\tau}}, \tag{80}$$

where we now denote the coefficients as $\hat{t}^{ii}_\tau$ since we now have $\hat{t}^{ii}_\tau \geqslant 0$, and where

$$\epsilon(\widehat{O}_i) = \text{Tr}\left[\widehat{O}_i^2\right] - \frac{\text{Tr}\left[\widehat{O}_i\right]^2}{2^m} = D_{HS}\left(\widehat{O}_i, \text{Tr}[\widehat{O}_i]\frac{\mathbb{1}}{2^m}\right). \tag{81}$$

Moreover, we also find that the following inequality holds $\forall i$

$$\sum_\tau \hat{t}^{ii}_\tau \geqslant \frac{2^{m(L-l)/2}}{(2^m+1)^{L-l}}, \tag{82}$$

where we recall that L is the number of layers in the ansatz, and that the block W is in the lth-layer of $V(\boldsymbol{\theta})$.

Combining Eqs. (71) and (80), we find

$$\text{Var}[\partial_\nu C] = \frac{2^{m-1}\text{Tr}[\sigma_\nu^2]}{(2^{2m}-1)^2}\sum_{\substack{\boldsymbol{pq}\\ \boldsymbol{p}'\boldsymbol{q}'}}\sum_{i\in i_{\mathcal{L}}} c_i^2\epsilon(\widehat{O}_i)\sum_\tau \hat{t}^{ii}_\tau \delta_{(\boldsymbol{p},\boldsymbol{q})_{S_{\overline{\mathcal{L}}}\cup S_{\overline{\tau}}}}\delta_{(\boldsymbol{p}',\boldsymbol{q}')_{S_{\overline{\mathcal{L}}}\cup S_{\overline{\tau}}}}\delta_{(\boldsymbol{p},\boldsymbol{q}')_{S_\tau}}\delta_{(\boldsymbol{p}',\boldsymbol{q})_{S_\tau}}\left\langle \Delta\Psi^{\boldsymbol{p}'\boldsymbol{q}'}_{\boldsymbol{pq}}\right\rangle_{V_{\text{L}}}. \tag{83}$$

Before proceeding to compute the expectation value over V_{L}, let us analyze the lower bound in (82). For simplicity of notation, let us define $T_\tau := \sum_\tau \hat{t}^{ii}_\tau$. In what follows we compare T_τ for two relevant (and extremum) cases: 1) when O_i acts nontrivially on the topmost m qubits in $V_{\mathcal{L}}$ (see panel (a) of Fig. (2)), and 2) when O_i acts nontrivially on S_w (see panel (b) of Fig. (2)). As discussed below, T_τ is larger for Case 2, that for Case 1.

As schematically depicted in Sup. Fig. (2)(a), for Case 1 one needs to integrate over $(L-l)$ blocks, and we know from Eq. (79) (and Sup. Fig. 1) that

$$T_\tau = \frac{2^{m(L-l)/2}}{(2^m+1)^{L-l}}. \tag{84}$$

On the other hand, for Case 2 one needs to integrate over $(L-l)(L-l+2)/4$ blocks. While a closed formula cannot be derived for this case, we can explicitly integrate over each block to obtain:

$$T_\tau = \begin{cases} \frac{2^{3m/2}(1+2^{m/2})^2}{(1+2^m)^3} & \text{if } L-l=2 \\ \frac{2^{2m}(1+2^{m/2})^2(1+2^{m/2}+2^m)}{1+d^2)^5} & \text{if } L-l=3 \\ \frac{2^{5m/2}(1+2^{m/2}+2^m)(1+2^{m/2}(3+2^{m/2}(8+2^{m/2}(3+2^{m/2}))))}{(1+2^m)^7} & \text{if } L-l=4 \\ \frac{2^{3m}(1+2^{m/2}(2+2^{m/2}(4+2^{m/2}(2+2^{m/2}))))(1+2^{m/2}(3+2^{m/2}(8+2^{m/2}(3+2^{m/2}))))}{(1+2^m)^9} & \text{if } L-l=5 \end{cases}. \tag{85}$$

For all cases we can see from Sup. Fig. (2)(c) that the values of T_τ for Case 2 are larger than the ones obtained for Case 1.

The latter can be understood from two key facts. First, that while integrating over a block leads to coefficients smaller than one according to (3), the more blocks one integrates over, the more contributions we get for $\epsilon(\widehat{O}_i)$.

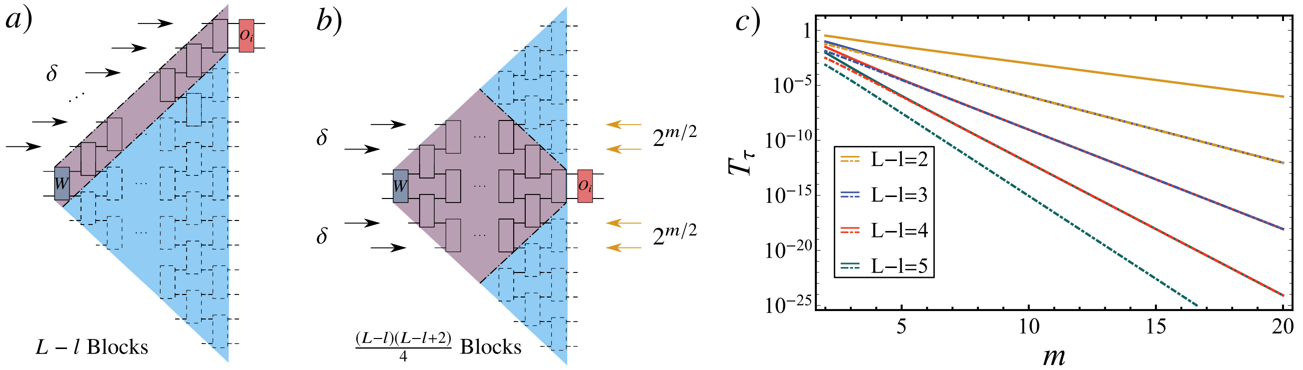

Supplementary Figure 2. a) Schematic representation of Case 1. The operator O_i acts nontrivially on the m topmost qubits in $V_{\mathcal{L}}$. Note that this scenario corresponds to the one analyzed in Sup. Fig. 1. Here one needs to integrate over $(L-l)$ blocks. The arrows indicate the tensor indexes that, when contracted after integrating over each block, will lead to delta functions in $\boldsymbol{p}$ and $\boldsymbol{q}$. b) Schematic representation of Case 2. The operator O_i acts non trivially on S_w. Here one needs to integrate over $(L-l)(L-l+2)/4$ blocks. For the case of L odd, the operator O_i acts nontrivially on the top $m/2$ qubits of S_w and the $m/2$ qubits above those. The arrows indicate the tensor indexes that, when contracted after integrating over each block, will lead to either delta functions in $\boldsymbol{p}$ and $\boldsymbol{q}$, or to traces over the identity on $m/2$ qubits. c) Solid lines represent the value of T_τ versus m for Case 2 in panel b) (i.e., from Eq. (85)). Dashed lines represent the value of T_τ versus m for Case 1 in panel a) (i.e., from Eq. (84)). The y axis is shown in a logarithmic scale. For all values of $(L-l)$ considered the values of T_τ Case 2 are larger that those Case 1. For a given value of $(L-l)$ the difference between values of T_τ in (84) and in (85) scales as $1/2^{m(L-l-1)/2}$. The previous explains why as m increases we the dashed line for $(L-l)$ converges to the solid line with $(L-l+1)$.

Second, from the fact that in Case 2 the integration over some blocks will lead to multiplicative factors $2^{m/2}$ which will compensate the factors from (3). These large factors $2^{m/2}$ essentially arise when integrating blocks in $V_{\mathcal{L}}$ which act on qubits which one does not measure, and which hence lead to traces over the identity over $m/2$ qubits. Note that these factors do not arise in Case I. In Sup. Fig. (2)(a) we have schematically indicated by arrows the indexes that will lead to delta functions in $\boldsymbol{p}$ and $\boldsymbol{q}$ after each block is integrated. Each time we integrate a block containing these indexes one obtains tensor contractions of $|\boldsymbol{p}\rangle\langle\boldsymbol{q}|$. On the other hand, for Case 2, one will not only get these tensor contractions in $\boldsymbol{p}$ and $\boldsymbol{q}$, but as indicated in Sup. Fig. (2)(b) we have also indexes that will lead to identity-tensor contractions and hence to factors $2^{m/2}$.

In fact, for a given L the difference between the T_τ in (84) and in (85) scales as $1/2^{m(L-l-1)/2}$, which means that as $(L-l)$ increases the inequality in (82) becomes tighter. Finally, we remark that the previous explains why in Fig. (2)(c) the values of T_τ from Case 1 and fixed $(L-l)$ converge to the values of Case 2 and $(L-l+1)$.

2. *Expectation value over* V_L

Let us now consider the term

$$\left\langle \Delta\Psi_{\boldsymbol{pq}}^{\boldsymbol{p'q'}} \right\rangle_{V_{\rm L}} = \left\langle \mathrm{Tr}[\Psi_{\boldsymbol{pq}}\Psi_{\boldsymbol{p'q'}}] - \frac{\mathrm{Tr}[\Psi_{\boldsymbol{pq}}]\mathrm{Tr}[\Psi_{\boldsymbol{p'q'}}]}{2^m} \right\rangle_{V_{\rm L}} . \tag{86}$$

Since the bitstring $\boldsymbol{p}$ ($\boldsymbol{q}$) can be expressed as a bit-wise concatenation of the form $\boldsymbol{p} = (\boldsymbol{p})_{S_{\overline{\mathcal{L}}}\cup S_{\overline{\tau}}} \cdot (\boldsymbol{p})_{S_\tau}$ (and similarly for $\boldsymbol{q}$), we can first evaluate

$$\sum_{(\boldsymbol{pq})_{S_{\overline{\mathcal{L}}}\cup S_{\overline{\tau}}}} \delta_{(\boldsymbol{p},\boldsymbol{q})_{S_{\overline{\mathcal{L}}}\cup S_{\overline{\tau}}}} \Psi_{\boldsymbol{qp}} = \mathrm{Tr}_\tau\left[(|\boldsymbol{p}\rangle\langle\boldsymbol{q}|_{S_\tau} \otimes \mathbb{1}_w)\tilde{\rho}_{Wk} \right] , \tag{87}$$

where Tr_τ is the partial trace over the Hilbert space $\mathcal{H}_\tau$ of the qubits in S_τ, and where we defined

$$\tilde{\rho}_{Wk} = \mathrm{Tr}_{\overline{\mathcal{L}}\cup\overline{\tau}}[V_{\rm L}\rho V_{\rm L}^\dagger] , \tag{88}$$

as the reduced state of $V_{\rm L}\rho V_{\rm L}^\dagger$ on $\mathcal{H}_w \otimes \mathcal{H}_\tau$. Then, from the term $\delta_{(\boldsymbol{p},\boldsymbol{q}')_{S_\tau}}\delta_{(\boldsymbol{p}',\boldsymbol{q})_{S_\tau}}$ in (83), we get

$$\sum_{(\boldsymbol{pq})_{S_\tau}} \delta_{(\boldsymbol{p},\boldsymbol{q}')_{S_\tau}}\delta_{(\boldsymbol{p}',\boldsymbol{q})_{S_\tau}} \left(\mathrm{Tr}[\Psi_{\boldsymbol{pq}}\Psi_{\boldsymbol{qp}}] - \frac{\mathrm{Tr}[\Psi_{\boldsymbol{pq}}]\mathrm{Tr}[\Psi_{\boldsymbol{qp}}]}{2^m} \right) = D_{HS}\left(\tilde{\rho}_{w\tau}, \tilde{\rho}_\tau \otimes \frac{\mathbb{1}}{2^m} \right) , \tag{89}$$

where $D_{HS}(\rho,\sigma) = \mathrm{Tr}[(\rho-\sigma)^2]$ is the Hilbert-Schmidt distance, and where we defined

$$\widetilde{\rho}_{\tau} = \mathrm{Tr}_{w}(\widetilde{\rho}_{w\tau}) \,, \quad \text{and} \quad \widetilde{\rho}_{w} = \mathrm{Tr}_{\tau}(\widetilde{\rho}_{w\tau}) = \mathrm{Tr}_{\overline{w}}\left[V_{\mathrm{L}}\rho V_{\mathrm{L}}^{\dagger}\right] \,, \tag{90}$$

as the reduces states of $V_{\mathrm{L}}\rho V_{\mathrm{L}}^{\dagger}$ on subsystem $\mathcal{H}_{\tau}$, and $\mathcal{H}_{w}$, respectively. Equation (89) quantifies how far $\widetilde{\rho}_{w\tau}$ is from being a tensor product state where the state on subsystem S_w is maximally mixed. Evidently, if $\widetilde{\rho}_{w}$ is maximally mixed then it will be impossible to train any angle in W.

Let us now analyze the following chain of inequalities valid for the Hilbert-Schmidt distance $D_{HS}\left(\widetilde{\rho}_{w\tau_t}, \widetilde{\rho}_{\tau_t} \otimes \frac{\mathbb{1}}{2^m}\right)$ and any choice of S_{τ}:

$$D_{HS}\left(\widetilde{\rho}_{w\tau_t}, \widetilde{\rho}_{\tau_t} \otimes \frac{\mathbb{1}}{2^m}\right) \geqslant \frac{4D_T\left(\widetilde{\rho}_{w\tau_t}, \widetilde{\rho}_{\tau_t} \otimes \frac{\mathbb{1}}{2^m}\right)^2}{2^m d_{\tau}} \tag{91}$$

$$\geqslant \frac{4D_T\left(\widetilde{\rho}_{w}, \frac{\mathbb{1}}{2^m}\right)^2}{2^{m(L-l+2)/2}}$$

$$\geqslant \frac{D_{HS}\left(\widetilde{\rho}_{w}, \frac{\mathbb{1}}{2^m}\right)}{2^{m(L-l+2)/2}} \,, \tag{92}$$

with $D_T(A,B) = \mathrm{Tr}\left[\sqrt{(A-B)^2}\right]$ the Trace Distance between the Hermitian operators A and B. In the first line we employ the matrix norm equivalence, and we denote as d_{τ} the dimension of $\mathcal{H}_{\tau}$. The second line is derived from the fact that the Trace Distance is non-increasing over partial trace [6], and from the fact that $d_{\tau} \leqslant 2^{m(L-l)/2}$ $\forall \tau$. Finally, the inequality in (92) employs again the matrix norm equivalence. From Eqs. (92), and (83), we find that the following lower bound holds

$$\mathrm{Var}[\partial_{\nu}C] \geqslant \frac{\mathrm{Tr}[\sigma_{\nu}^2]}{2(2^{2m}-1)^2(2^m+1)^{L-l}} \sum_{i\in i_{\mathcal{L}}} c_i^2 \epsilon_i \left\langle D_{HS}\left(\widetilde{\rho}_{w}, \frac{\mathbb{1}}{2^m}\right)\right\rangle_{V_{\mathrm{L}}} \,. \tag{93}$$

Following a similar procedure as the one previously employed to compute expectation values over V_{R}, we can once again leverage the tensor network representation of quantum circuits to algorithmically integrate over each block and compute $\langle D_{HS}\left(\widetilde{\rho}_{W}, \frac{\mathbb{1}}{2^m}\right)\rangle_{V_{\mathrm{L}}}$. After considering that all the blocks in V_{L} which are not in the backpropagated light-cone of W will simplify to identity, we find

$$\left\langle D_{HS}\left(\widetilde{\rho}_{w}, \frac{\mathbb{1}}{2^m}\right)\right\rangle_{V_{\mathrm{L}}} = \sum_{\substack{(k,k')\in k_{\mathcal{L}_{\mathrm{B}}} \\ k'\geqslant k}} t_{k,k'}\epsilon(\rho_{k,k'}) \,, \tag{94}$$

where $t_{k,k'} \geqslant 0$, and where $k_{\mathcal{L}_{\mathrm{B}}}$ is the set of k indices whose associated subsystems S_k are in the backward light-cone $\mathcal{L}_{\mathrm{B}}$ of W. Here we defined the function $\epsilon(M) = D_{\mathrm{HS}}\left(M, \mathrm{Tr}(M)\mathbb{1}/d_M\right)$ where D_{HS} is the Hilbert-Schmidt distance and d_M is the dimension of the matrix M. In addition, $\rho_{k,k'}$ is partial trace of the input state ρ down to the subsystems $S_k S_{k+1} \ldots S_{k'}$. In addition, we find that the following inequality holds $\forall k,k'$

$$t_{k,k'} \geqslant \frac{2^{ml}}{(2^m+1)^{2l}} \,. \tag{95}$$

Hence, we have $\mathrm{Var}[\partial_{\nu}C] \geqslant G_n(L,l)$, where

$$G_n(L,l) = \frac{2^{m(l+1)-1}}{(2^{2m}-1)^2(2^m+1)^{L+l}} \sum_{i\in i_{\mathcal{L}}} \sum_{\substack{(k,k')\in k_{\mathcal{L}_{\mathrm{B}}} \\ k'\geqslant k}} c_i^2 \epsilon(\rho_{k,k'})\epsilon(\widehat{O}_i) \,, \tag{96}$$

where we used the fact that $\mathrm{Tr}[\sigma_{\nu}^2] = 2^m$.

This result can be trivially generalized to the case when $\widehat{O}_i$ and $\widehat{O}_j$ are of the general form in Eq. (68) such that they can have overlapping support on at most $m/2$ qubits. Then, from (77) it is straightforward to see that $\Delta O_{\tau}^{ij} = 0$ for all τ, i, and j, as one would always have to compute traces of the form $\mathrm{Tr}[\widehat{O}_i^{\mu_i}]$, which vanish since $\widehat{O}_i^{\mu_i}$ can be written as a tensor product of Pauli operators.

□

Supplementary Note 7: Proof of Theorem 1

The following theorem provides an upper bound on the variance of the partial derivative of a global cost function which can be expressed as the expectation value of an operator of the form

$$O = c_0 \mathbb{1} + \sum_{i=1}^{N} c_i \widehat{O}_{i1} \otimes \widehat{O}_{i2} \otimes \cdots \otimes \widehat{O}_{i\xi} \,. \tag{97}$$

Specifically, we consider two cases of interest: (i) When $N = 1$ and each $\widehat{O}_{1k}$ is a non-trivial projector ($\widehat{O}_{1k}^2 = \widehat{O}_{1k} \neq \mathbb{1}$) of rank r_k acting on subsystem S_k, or (ii) When N is arbitrary and $\widehat{O}_{ik}$ is traceless with $\mathrm{Tr}[\widehat{O}_{ik}^2] \leqslant 2^m$ (for example, when $\widehat{O}_{ik} = \bigotimes_{j=1}^{m} \sigma_j^\mu$ is a tensor product of Pauli operators $\sigma_j^\mu \in \{\mathbb{1}_j, \sigma_j^x, \sigma_j^y, \sigma_j^z\}$, with at least one $\sigma_j^\mu \neq \mathbb{1}$). In Section 7 A we first consider case (i), while in Section 7 B we prove Theorem 1 for case (ii).

We now reiterate Theorem 1 for convenience:

Theorem 1. Consider a trainable parameter θ^ν in a block W of the ansatz in Fig. 3 of the main text. Let $\mathrm{Var}[\partial_\nu C]$ be the variance of the partial derivative of a global cost function C (with O given by (97)) with respect to θ^ν. If W_{A}, W_{B} of (39), and each block in $V(\boldsymbol{\theta})$ form a local 2-design, then $\mathrm{Var}[\partial_\nu C]$ is upper bounded by

$$\mathrm{Var}[\partial_\nu C] \leqslant F_n(L, l) \,. \tag{98}$$

(i) For $N = 1$ and when each $\widehat{O}_{1k}$ is a non-trivial projector, then defining $R = \prod_{k=1}^{\xi} r_k^2$, we have

$$F_n(L, l) = \frac{2^{2m+(2m-1)(L-l)}}{(2^{2m}-1) \cdot 3^{\frac{n}{m}} \cdot 2^{(2-\frac{3}{m})n}} c_1^2 R \,. \tag{99}$$

(ii) For arbitrary N and when each $\widehat{O}_{ik}$ satisfies $\mathrm{Tr}[\widehat{O}_{ik}] = 0$ and $\mathrm{Tr}[\widehat{O}_{ik}^2] \leqslant 2^m$, then

$$F_n(L, l) = \frac{2^{2m(L-l+1)+1}}{3^{\frac{2n}{m}} \cdot 2^{\left(3-\frac{4}{m}\right)n}} \sum_{i,j=1}^{N} c_i c_j \,. \tag{100}$$

A. $N = 1$, and $\widehat{O}_{1k}$ is a non-trivial projector

For simplicity, let us now use $\widehat{O}_k$ when refering to the operators $\widehat{O}_{ik}$. Let us first consider the case when each $\widehat{O}_k$ is a non-trivial projector ($\widehat{O}_k^2 = \widehat{O}_k$) of rank r_k acting on subsystem S_k:

$$r_k := \mathrm{Tr}\left[\widehat{O}_k\right] = \mathrm{Tr}\left[\widehat{O}_k^2\right] = \mathrm{rank}\left[\widehat{O}_k\right] \,. \tag{101}$$

Proof. In the previous section, we defined V_{R} as containing all gates in the forward light-cone $\mathcal{L}$ of W and all the gates in the last layer of the ansatz. Hence, we can express $V_{\mathrm{R}} = V_{\mathcal{L}} \otimes V_{\overline{\mathcal{L}}}$, where $V_{\overline{\mathcal{L}}} : \mathcal{H}_{\overline{\mathcal{L}}} \to \mathcal{H}_{\overline{\mathcal{L}}}$ is given by

$$V_{\overline{\mathcal{L}}} = \bigotimes_{k \in k_{\overline{\mathcal{L}}}} W_{kL} \,, \tag{102}$$

where where $k_{\overline{\mathcal{L}}}$ is the set of k indices whose associated subsystems of qubits S_k are outside of the forward light-cone $\mathcal{L}$ of W. One can always write $|\boldsymbol{q}\rangle\langle\boldsymbol{p}| \otimes \mathbb{1}_w$ as a projector onto $H_{\mathcal{L}}$ times a projector onto $H_{\overline{\mathcal{L}}}$:

$$|\boldsymbol{q}\rangle\langle\boldsymbol{p}| \otimes \mathbb{1}_w = \left(\bigotimes_{k \in k_{\overline{\mathcal{L}}}} |\boldsymbol{q}\rangle\langle\boldsymbol{p}|_k \right) \otimes |\boldsymbol{q}\rangle\langle\boldsymbol{p}|_{\mathcal{L}} \otimes \mathbb{1}_w \,. \tag{103}$$

Combining (49) with Eqs. (102) and (103) leads to

$$\Omega_{\boldsymbol{qp}} = \left(\prod_{k \in k_{\overline{\mathcal{L}}}} \mathrm{Tr}_k \left[|\boldsymbol{p}\rangle\langle\boldsymbol{q}|_k W_{kL}^\dagger \widehat{O}_k W_{kL} \right] \right) \Omega_{\boldsymbol{qp}}^{\mathcal{L}} \,, \tag{104}$$

where

$$\Omega_{\boldsymbol{qp}}^{\mathcal{L}} = \text{Tr}_{\mathcal{L}\cap\overline{w}}\left[(|\boldsymbol{p}\rangle\langle\boldsymbol{q}|\otimes \mathbb{1}_w)V_{\mathcal{L}}O^{\mathcal{L}}V_{\mathcal{L}}\right] , \tag{105}$$

and where $O^{\mathcal{L}} = \bigotimes_{k\in k_{\overline{\mathcal{L}}}} \widehat{O}_k$. Here Tr_k indicates the trace over $\mathcal{H}_k$, while $\text{Tr}_{\mathcal{L}\cap\overline{w}}$ is the trace over the Hilbert space associated with the qubits in $S_{\mathcal{L}}\cap S_{\overline{w}}$.

In order to explicitly evaluate the expectation value $\langle\ldots\rangle_{V_{\text{R}}}$ in

$$\text{Var}[\partial_\nu C] = \frac{2^{m-1}\text{Tr}[\sigma_\nu^2]}{(2^{2m}-1)^2}c_1^2\sum_{\substack{\boldsymbol{pq}\\ \boldsymbol{p'q'}}}\left\langle\left(\text{Tr}[\Omega_{\boldsymbol{qp}}\Omega_{\boldsymbol{q'p'}}] - \frac{\text{Tr}[\Omega_{\boldsymbol{qp}}]\text{Tr}[\Omega_{\boldsymbol{q'p'}}]}{2^m}\right)\right\rangle_{V_{\text{R}}}\left\langle\Delta\Psi_{\boldsymbol{pq}}^{\boldsymbol{p'q'}}\right\rangle_{V_{\text{L}}} , \tag{106}$$

we use the fact that the blocks in $V(\boldsymbol{\theta})$ are independent, and hence $\langle\ldots\rangle_{V_{\text{R}}} = \langle\ldots\rangle_{V_{\overline{\mathcal{L}}},V_{\mathcal{L}}}$. Then, we have

$$\left\langle\text{Tr}[\Omega_{\boldsymbol{qp}}\Omega_{\boldsymbol{q'p'}}] - \frac{\text{Tr}[\Omega_{\boldsymbol{qp}}]\text{Tr}[\Omega_{\boldsymbol{q'p'}}]}{2^m}\right\rangle_{V_{\text{R}}} = \left\langle\text{Tr}[\Omega_{\boldsymbol{qp}}^{\mathcal{L}}\Omega_{\boldsymbol{q'p'}}^{\mathcal{L}}] - \frac{\text{Tr}[\Omega_{\boldsymbol{qp}}^{\mathcal{L}}]\text{Tr}[\Omega_{\boldsymbol{q'p'}}^{\mathcal{L}}]}{2^m}\right\rangle_{V_{\mathcal{L}}}\left(\prod_{k/S_k\subset S_{\overline{\mathcal{L}}}}\langle\Omega_k\rangle_{W_{kL}}\right) , \tag{107}$$

with

$$\Omega_k = \text{Tr}_k\left[|\boldsymbol{p}\rangle\langle\boldsymbol{q}|_k W_{kL}^\dagger\widehat{O}_k W_{kL}\right]\text{Tr}_k\left[|\boldsymbol{p'}\rangle\langle\boldsymbol{q'}|_k W_{kL}^\dagger\widehat{O}_k W_{kL}\right] . \tag{108}$$

Let us first compute the expectation value of each Ω_k. From Lemma 3, we have:

$$\begin{aligned}\langle\Omega_k\rangle_{W_{kL}} &= \frac{r_k}{2^{2m}-1}\left(\left(r_k - \frac{1}{2^m}\right)\delta_{(\boldsymbol{p},\boldsymbol{q})_{S_k}}\delta_{(\boldsymbol{p'},\boldsymbol{q'})_{S_k}} + \left(1-\frac{r_k}{2^m}\right)\delta_{(\boldsymbol{p},\boldsymbol{q'})_{S_k}}\delta_{(\boldsymbol{p'},\boldsymbol{q})_{S_k}}\right)\\ &\leqslant \frac{1}{2^{2m}-1}r_k^2\left(\delta_{(\boldsymbol{p},\boldsymbol{q})_{S_k}}\delta_{(\boldsymbol{p'},\boldsymbol{q'})_{S_k}} + \delta_{(\boldsymbol{p},\boldsymbol{q'})_{S_k}}\delta_{(\boldsymbol{p'},\boldsymbol{q})_{S_k}}\right) ,\end{aligned} \tag{109}$$

where in the inequality we have dropped the negative terms and used the fact that $r_k \leqslant r_k^2$. Then, we have

$$\prod_{k\in k_{\overline{\mathcal{L}}}}\langle\Omega_k\rangle_{W_{kL}} \leqslant \frac{1}{(2^{2m}-1)^{\xi_{\overline{\mathcal{L}}}}}\prod_{k\in k_{\overline{\mathcal{L}}}} r_k^2\left(\delta_{(\boldsymbol{p},\boldsymbol{q})_{S_k}}\delta_{(\boldsymbol{p'},\boldsymbol{q'})_{S_k}} + \delta_{(\boldsymbol{p},\boldsymbol{q'})_{S_k}}\delta_{(\boldsymbol{p'},\boldsymbol{q})_{S_k}}\right) .$$

Combining this result with Eq. (106) leads to the upper bound

$$\begin{aligned}\text{Var}[\partial_\nu C] \leqslant{}& \frac{2^{m-1}\text{Tr}[\sigma_\nu^2]}{(2^{2m}-1)^2(2^{2m}-1)^{\xi_{\overline{\mathcal{L}}}}}c_1^2\sum_{\substack{\boldsymbol{pq}\\ \boldsymbol{p'q'}}}\left(\left\langle\text{Tr}[\Omega_{\boldsymbol{qp}}^{\mathcal{L}}\Omega_{\boldsymbol{q'p'}}^{\mathcal{L}}] - \frac{\text{Tr}[\Omega_{\boldsymbol{qp}}^{\mathcal{L}}]\text{Tr}[\Omega_{\boldsymbol{q'p'}}^{\mathcal{L}}]}{2^m}\right\rangle_{V_{\mathcal{L}}}\right.\\ &\left.\times\left(\prod_{k\in k_{\overline{\mathcal{L}}}} r_k^2\left(\delta_{(\boldsymbol{p},\boldsymbol{q})_{S_k}}\delta_{(\boldsymbol{p'},\boldsymbol{q'})_{S_k}} + \delta_{(\boldsymbol{p},\boldsymbol{q'})_{S_k}}\delta_{(\boldsymbol{p'},\boldsymbol{q})_{S_k}}\right)\right)\left\langle\Delta\Psi_{\boldsymbol{pq}}^{\boldsymbol{p'q'}}\right\rangle_{V_{\text{L}}}\right) .\end{aligned} \tag{110}$$

As discussed in the previous section, one can compute the expectation values in Eq. (110) by systematically integrating over each block over the unitary group with the respect to the Haar measure. From Eq. (76) we can find

$$\begin{aligned}\text{Var}[\partial_\nu C] \leqslant{}& \frac{2^{m-1}\text{Tr}[\sigma_\nu^2]}{(2^{2m}-1)^2(2^{2m}-1)^{\xi_{\overline{\mathcal{L}}}}}c_1^2\sum_{\substack{\boldsymbol{pq}\\ \boldsymbol{p'q'}}}\sum_{\tau}\left(t_\tau\delta_{(\boldsymbol{p},\boldsymbol{q})_{S_{\overline{\tau}}}}\delta_{(\boldsymbol{p'},\boldsymbol{q'})_{S_{\overline{\tau}}}}\delta_{(\boldsymbol{p},\boldsymbol{q'})_{S_\tau}}\delta_{(\boldsymbol{p'},\boldsymbol{q})_{S_\tau}}\Delta O_\tau\right.\\ &\left.\times\left(\prod_{k\in k_{\overline{\mathcal{L}}}} r_k^2\left(\delta_{(\boldsymbol{p},\boldsymbol{q})_{S_k}}\delta_{(\boldsymbol{p'},\boldsymbol{q'})_{S_k}} + \delta_{(\boldsymbol{p},\boldsymbol{q'})_{S_k}}\delta_{(\boldsymbol{p'},\boldsymbol{q})_{S_k}}\right)\right)\left\langle\Delta\Psi_{\boldsymbol{pq}}^{\boldsymbol{p'q'}}\right\rangle_{V_{\text{L}}}\right) .\end{aligned} \tag{111}$$

Note that by expanding the product $\prod_{k\in k_{\overline{\mathcal{L}}}}\left(\delta_{(\boldsymbol{p},\boldsymbol{q})_{S_k}}\delta_{(\boldsymbol{p'},\boldsymbol{q'})_{S_k}} + \delta_{(\boldsymbol{p},\boldsymbol{q'})_{S_k}}\delta_{(\boldsymbol{p'},\boldsymbol{q})_{S_k}}\right)\delta_{(\boldsymbol{p},\boldsymbol{q})_{S_{\overline{\tau}}}}\delta_{(\boldsymbol{p'},\boldsymbol{q'})_{S_{\overline{\tau}}}}\delta_{(\boldsymbol{p},\boldsymbol{q'})_{S_\tau}}\delta_{(\boldsymbol{p'},\boldsymbol{q})_{S_\tau}}$, one obtains a sum of $2^{\xi_{\overline{\mathcal{L}}}}$ terms, and according to Eqs. (86)–(89), each term in the summation leads to a Hilbert-Schmidt distances between two quantum states. Then, since $D_{HS}(\rho_1,\rho_2)\leqslant 2$ for any pair of states ρ_1, ρ_2, we find

$$\sum_{\substack{\boldsymbol{pq}\\ \boldsymbol{p'q'}}}\delta_{(\boldsymbol{p},\boldsymbol{q})_{S_{\overline{\tau}}}}\delta_{(\boldsymbol{p'},\boldsymbol{q'})_{S_{\overline{\tau}}}}\delta_{(\boldsymbol{p},\boldsymbol{q'})_{S_\tau}}\delta_{(\boldsymbol{p'},\boldsymbol{q})_{S_\tau}}\left(\prod_{k\in k_{\overline{\mathcal{L}}}}\left(\delta_{(\boldsymbol{p},\boldsymbol{q})_{S_k}}\delta_{(\boldsymbol{p'},\boldsymbol{q'})_{S_k}} + \delta_{(\boldsymbol{p},\boldsymbol{q'})_{S_k}}\delta_{(\boldsymbol{p'},\boldsymbol{q})_{S_k}}\right)\right)\left\langle\Delta\Psi_{\boldsymbol{pq}}^{\boldsymbol{p'q'}}\right\rangle_{V_{\text{L}}} \leqslant 2\cdot 2^{\xi_{\overline{\mathcal{L}}}} . \tag{112}$$

Replacing this result in (111) leads to

$$\mathrm{Var}[\partial_\nu C] \leqslant \frac{2^m 2^{\xi_{\overline{\mathcal{L}}}} \mathrm{Tr}[\sigma_\nu^2]}{(2^m-1)^2(2^{2m}-1)^{\xi_{\overline{\mathcal{L}}}}} c_1^2 \left(\prod_{k\in k_{\overline{\mathcal{L}}}} r_k^2\right) \sum_\tau t_\tau \Delta O_\tau \,. \tag{113}$$

Next, we consider the terms ΔO_τ. From Eq. (77) we can show that

$$\Delta O_\tau \leqslant \prod_{k\in k_{\mathcal{L}}} r_k^2 \,, \tag{114}$$

so that (113) becomes

$$\mathrm{Var}[\partial_\nu C] \leqslant \frac{2^m 2^{\xi_{\overline{\mathcal{L}}}} \mathrm{Tr}[\sigma_\nu^2]}{(2^{2m}-1)^2(2^{2m}-1)^{\xi_{\overline{\mathcal{L}}}}} c_1^2 R \sum_\tau t_\tau \,, \tag{115}$$

where $R = \prod_{k=1}^{\xi} r_k^2$.

Let us finally show that $\sum_\tau t_\tau \leqslant 2\ \forall l, L$. We recall from Eq. (106) that the coefficients t_τ are obtained by integrating each block in $V(\boldsymbol{\theta})$ over the unitary group with the respect to the Haar measure. Each time a block is integrated one obtains four new tensors weighted by the coefficients η_i, with $i=1,\dots,4$ such that $\sum_{\mu=1}^4 |\eta_\mu| \leqslant 1$ for all m. Consider now the average $\left\langle \mathrm{Tr}[\Omega_{\boldsymbol{qp}}^i \Omega_{\boldsymbol{q'p'}}^j]\right\rangle_{V_{\mathrm{R}}}$, once all the blocks have been integrated we find

$$\left\langle \mathrm{Tr}[\Omega_{\boldsymbol{qp}}^i \Omega_{\boldsymbol{q'p'}}^j]\right\rangle_{V_{\mathrm{R}}} = \sum_{\mu_1=1}^4 \eta_{\mu_1}\left(\sum_{\mu_2=1}^4 \eta_{\mu_2}\left(\dots\left(\sum_{\mu_m=1}^4 \eta_{\mu_m}\right)\right)\right) T_{\mu_1,\mu_2,\dots,\mu_m}(O)\,, \tag{116}$$

where μ_m is the number of averaged blocks, and where $T_{\mu_1,\mu_2,\dots,\mu_m}(O)$ is a tensor contraction of O. A similar equation can be obtained for $\left\langle \mathrm{Tr}[\Omega_{\boldsymbol{qp}}^i]\mathrm{Tr}[\Omega_{\boldsymbol{q'p'}}^j]\right\rangle_{V_{\mathrm{R}}}$ as

$$\left\langle \mathrm{Tr}[\Omega_{\boldsymbol{qp}}^i]\mathrm{Tr}[\Omega_{\boldsymbol{q'p'}}^j]\right\rangle_{V_{\mathrm{R}}} = \sum_{\mu'_1=1}^4 \eta_{\mu'_1}\left(\sum_{\mu'_2=1}^4 \eta_{\mu'_2}\left(\dots\left(\sum_{\mu'_m=1}^4 \eta_{\mu'_m}\right)\right)\right) T_{\mu'_1,\mu'_2,\dots,\mu'_m}(O)\,, \tag{117}$$

so that

$$\sum_\tau t_\tau = \sum_{\mu_1=1}^4 \eta_{\mu_1}\left(\sum_{\mu_2=1}^4 \eta_{\mu_2}\left(\dots\left(\sum_{\mu_m=1}^4 \eta_{\mu_m}\right)\right)\right) - \frac{1}{2^m}\sum_{\mu'_1=1}^4 \eta_{\mu'_1}\left(\sum_{\mu'_2=1}^4 \eta_{\mu'_2}\left(\dots\left(\sum_{\mu'_m=1}^4 \eta_{\mu'_m}\right)\right)\right)\,. \tag{118}$$

Taking the absolute value on both side and using the fact that $\sum_{\mu=1}^4 |\eta_\mu| \leqslant 1$, one gets $\sum_\tau t_\tau = |\sum_\tau t_\tau| \leqslant 1 + \frac{1}{2^m} \leqslant 2$.

Therefore, by using $\mathrm{Tr}\left[\sigma_\nu^2\right] = 2^m$, we have

$$\mathrm{Var}[\partial_\nu C] \leqslant \frac{2^{2m+\frac{n}{m}+l-L}}{(2^{2m}-1)^2(2^{2m}-1)^{\frac{n}{m}+l-L-1}} c_1^2 R\,.$$

Moreover, we also find that

$$\frac{2^{2m+\frac{n}{m}+l-L}}{(2^{2m}-1)^2(2^{2m}-1)^{\frac{n}{m}+l-L-1}} c_1^2 R = \frac{2^{2m}}{2^{2m}-1}\cdot\left(2^{2m-1}-\frac{1}{2}\right)^{L-l}\cdot\frac{2^{\frac{n}{m}}}{2^{2n}(1-2^{-2m})^{\frac{n}{m}}} c_1^2 R\,, \tag{119}$$

and since $2^{2m-1}-\frac{1}{2} < 2^{2m-1}$ and $1 \leqslant \frac{1}{1-2^{-2m}} \leqslant \frac{4}{3}$, $\mathrm{Var}[\partial_\nu C]$ can be upper bounded as $\mathrm{Var}[\partial_\nu C] < F_n(L,l)$, where

$$F_n(L,l) = \frac{2^{2m+(2m-1)(L-l)}}{(2^{2m}-1)\cdot 3^{\frac{n}{m}}\cdot 2^{(2-\frac{3}{m})n}} c_1^2 R\,. \tag{120}$$

□

B. Arbitrary N and $\widehat{O}_{ik}$ is a traceless operators such that $\mathrm{Tr}[\widehat{O}_{ik}^2] \leqslant 2^m$

We now consider the case when $\widehat{O}_{ik}$ is traceless. Specifically, we assume

$$\mathrm{Tr}\left[\widehat{O}_{ik}\right] = 0\,, \quad \text{and} \quad \mathrm{Tr}\left[\widehat{O}_{ik}^2\right] \leqslant 2^m\,. \tag{121}$$

Note that if $\widehat{O}_{ik}$ is a tensor product of Pauli operators then $\mathrm{Tr}\left[\widehat{O}_{ik}^2\right] = 2^m$.

Proof. Let us first analyze the case of $N = 1$. Combining Lemma 3 with Eqs. (107), and (108), we find

$$\langle \Omega_k \rangle_{W_{kL}} \leqslant \frac{2^m}{2^{2m}-1}\left(\delta_{(\boldsymbol{p'},\boldsymbol{q})_{S_k}}\delta_{(\boldsymbol{p},\boldsymbol{q'})_{S_k}} - \frac{1}{2^m}\delta_{(\boldsymbol{p},\boldsymbol{q})_{S_k}}\delta_{(\boldsymbol{p'},\boldsymbol{q'})_{S_k}}\right) \leqslant \frac{2^m}{2^{2m}-1}\delta_{(\boldsymbol{p'},\boldsymbol{q})_{S_k}}\delta_{(\boldsymbol{p},\boldsymbol{q'})_{S_k}}\,, \tag{122}$$

which yields

$$\prod_{k\in k_{\overline{\mathcal{L}}}} \langle \Omega_k \rangle_{W_{kL}} \leqslant \frac{2^{\xi_{\overline{\mathcal{L}}} m}}{(2^{2m}-1)^{\xi_{\overline{\mathcal{L}}}}} \prod_{k\in k_{\overline{\mathcal{L}}}} \delta_{(\boldsymbol{p'},\boldsymbol{q})_{S_k}}\delta_{(\boldsymbol{p},\boldsymbol{q'})_{S_k}}\,. \tag{123}$$

Therefore, from (111), and (123), we can obtain

$$\mathrm{Var}\left[\partial_\nu C\right] \leqslant \frac{2^{2m-1}}{(2^{2m}-1)^2(2^{2m}-1)^{\xi_{\mathcal{L}}}}c_1^2 \cdot \frac{2^{\xi_{\overline{\mathcal{L}}} m}}{(2^{2m}-1)^{\xi_{\overline{\mathcal{L}}}}} \tag{124}$$

$$\times \sum_{\substack{\boldsymbol{p}\boldsymbol{q}\\ \boldsymbol{p'}\boldsymbol{q'}}}\sum_{\tau} t_\tau \Delta O_\tau \left(\prod_{k\in k_{\overline{\mathcal{L}}}} \delta_{(\boldsymbol{p'},\boldsymbol{q})_{S_k}}\delta_{(\boldsymbol{p},\boldsymbol{q'})_{S_k}}\right) \delta_{(\boldsymbol{p},\boldsymbol{q})_{S_{\overline{\tau}}}}\delta_{(\boldsymbol{p'},\boldsymbol{q'})_{S_{\overline{\tau}}}}\delta_{(\boldsymbol{p},\boldsymbol{q'})_{S_\tau}}\delta_{(\boldsymbol{p'},\boldsymbol{q})_{S_\tau}} \left\langle \Delta \Psi_{\boldsymbol{pq}}^{\boldsymbol{p'q'}}\right\rangle_{V_{\mathrm{L}}}\,. \tag{125}$$

Let us now consider the terms ΔO_τ from (77). It is straightforward to see that $\Delta O_\tau = 0$ if $S_{\tau_z} \neq \emptyset$, due to the fact that $\mathrm{Tr}[\widehat{O}_{ik}] = 0$. Hence, let us define τ_p as the set of indexes such that $S_{\tau_z} = \emptyset$. Then, we have

$$\Delta O_\tau \leqslant \begin{cases} 2^{m(L-l+1)} & (\tau \in \tau_p) \\ 0 & (\tau \notin \tau_p) \end{cases}\,. \tag{126}$$

The latter implies

$$\sum_\tau t_\tau \Delta O_\tau \leqslant \sum_{\tau\in\tau_p} t_\tau \Delta O_\tau \leqslant 2 \times 2^{m(L-l+1)}\,, \tag{127}$$

where we used the fact that $\sum t_\tau \leqslant 2$ (see Eq. (118) in the previous section).

According to Eqs. (86)–(89), the product $\left(\prod_{k\in k_{\overline{\mathcal{L}}}} \delta_{(\boldsymbol{p'},\boldsymbol{q})_{S_k}}\delta_{(\boldsymbol{p},\boldsymbol{q'})_{S_k}}\right)\delta_{(\boldsymbol{p},\boldsymbol{q})_{S_{\overline{\tau}}}}\delta_{(\boldsymbol{p'},\boldsymbol{q'})_{S_{\overline{\tau}}}}\delta_{(\boldsymbol{p},\boldsymbol{q'})_{S_\tau}}\delta_{(\boldsymbol{p'},\boldsymbol{q})_{S_\tau}}$, leads to a Hilbert-Schmidt distances between two quantum states, which is always upper bounded by 2. We then find

$$\mathrm{Var}\left[\partial_\nu C\right] \leqslant \frac{2^{2m+1}}{(2^{2m}-1)^2(2^{2m}-1)^{\xi_{\mathcal{L}}}}c_1^2 \cdot \frac{2^{\xi_{\overline{\mathcal{L}}} m}}{(2^{2m}-1)^{\xi_{\overline{\mathcal{L}}}}} \cdot 2^{m(L-l+1)}\,. \tag{128}$$

Since $n = m(L-l+1) + m\xi_{\overline{\mathcal{L}}}$, we can rewrite (128) as

$$\mathrm{Var}\left[\partial_\nu C\right] \leqslant \frac{2^{2m+n+1}(2^{2m}-1)^{2(L-l)}}{2^{4n}(1-2^{-2m})^{\frac{2n}{m}}}c_1^2\,. \tag{129}$$

Finally, noting that $2^{2m}-1 \leqslant 2^{2m}$ and $1 \leqslant \frac{1}{1-2^{-2m}} \leqslant \frac{4}{3}$, we have

$$\mathrm{Var}\left[\partial_\nu C\right] \leqslant \frac{2^{2m(L-l+1)+1}}{3^{\frac{2n}{m}}2^{(3-\frac{4}{m})n}}c_1^2\,. \tag{130}$$

Let us now consider the arbitrary N case, where the operator O can be expressed as

$$O = c_0 \mathbb{1} + \sum_{i=1}^N c_i O_i\,, \tag{131}$$

and where each O_i can be expressed as $O_i = \widehat{O}_{i1} \otimes \widehat{O}_{i2} \otimes \cdots \otimes \widehat{O}_{i\xi}$ with $\widehat{O}_{ik}$ satisying (121).

Here it is convinient to define $C_i = \text{Tr}[O_i V(\boldsymbol{\theta})\rho V^\dagger(\boldsymbol{\theta})]$, so that $C = c_0 + \sum_i c_i C_i$. When computing the variance we now have to consider the cross terms arising from C_i, and C_j with $i \neq j$:

$$\text{Var}[\partial_\nu C] = \langle (\partial_\nu C)^2 \rangle_V = \sum_i c_i^2 \langle (\partial_\nu C_i)^2 \rangle_V + \sum_{i\neq j} c_i c_j \langle \partial_\nu C_i \partial_\nu C_j \rangle_V \,. \tag{132}$$

First, let us remark that

$$\text{Tr}\left[\widehat{O}_{ik}\right] = 0\,, \quad \text{and} \quad \text{Tr}\left[\widehat{O}_{ik}\widehat{O}_{jk'}\right] \leqslant 2^m\,, \ \forall i,j,k,k' \quad \text{and} \quad \begin{cases} \Delta O_\tau^{ij} \leqslant 2^{m(L-l+1)} & (\tau \in \tau_p) \\ \Delta O_\tau^{ij} = 0 & (\tau \notin \tau_p) \end{cases}\,, \tag{133}$$

where ΔO_τ^{ij} was defined in (77), and where $\text{Tr}\left[\widehat{O}_{ik}\widehat{O}_{jk'}\right] \leqslant 2^m$ follows from the fact that $\text{Tr}\left[\widehat{O}_{ik}^2\right] \leqslant 2^m$ and $\text{Tr}\left[\widehat{O}_{jk'}^2\right] \leqslant 2^m$. Hence, it is straigtforward to see that the upper bound in Eq. (130) also holds for the cross terms in (132), and we then have

$$\text{Var}[\partial_\nu C] \leqslant F_n(L,l)\,. \tag{134}$$

where

$$F_n(L,l) = \frac{2^{2m(L-l+1)+1}}{3^{\frac{2n}{m}} 2^{\left(3-\frac{4}{m}\right)n}} \sum_{ij} c_i c_j\,. \tag{135}$$

□

Supplementary Note 8: Proofs of Corollaries

A. Proof of Corollary 1

Corollary 1. Consider the function $F_n(L,l)$.

(i) Let $N=1$ and let each $\widehat{O}_{1k}$ be a non-trivial projector, as in case (i) of Theorem 1. If $c_1^2 R \in \mathcal{O}(2^n)$ and if the number of layers $L \in \mathcal{O}(\text{poly}(\log(n)))$, then

$$F_n(L,l) \in \mathcal{O}\left(2^{-\left(1-\frac{1}{m}\log_2 3\right)n}\right)\,, \tag{136}$$

which implies that $\text{Var}[\partial_\nu C]$ is exponentially vanishing in n if $m \geqslant 2$.

(ii) Let N be arbitrary, and let each $\widehat{O}_{ik}$ satisfy $\text{Tr}[\widehat{O}_{ik}] = 0$ and $\text{Tr}[\widehat{O}_{ik}^2] \leqslant 2^m$, as in case (ii) of Theorem 1. If $N \in \mathcal{O}(2^n)$, $c_i \in \mathcal{O}(1)$, and if the number of layers $L \in \mathcal{O}(\text{poly}(\log(n)))$, then

$$F_n(L,l) \in \mathcal{O}\left(\frac{1}{2^{\left(1-\frac{1}{m}\right)n}}\right)\,, \tag{137}$$

which implies that $\text{Var}[\partial_\nu C]$ is exponentially vanishing in n if $m \geqslant 2$.

Let us first consider case (i) in Theorem 1.

Proof. Let us assume $L \in \mathcal{O}(\text{poly}(\log(n)))$, so that we have $2^{(2m-1)(L-l)} \in \mathcal{O}\left(2^{\text{poly}(\log(n))}\right)$. Here, note that

$$\lim_{n\to\infty}\left|\frac{\text{poly}(\log(n))}{n}\right| = 0,$$

which means that n grows faster than the any polylogarithmic functions of n. Therefore, we can write $\mathcal{O}(\text{poly}(\log(n))) \subset \mathcal{O}(n)$. For a nonzero constant a, we can write $\mathcal{O}(|a|n) = \mathcal{O}(n)$, and we can choose $a = \frac{1}{m}\log_2(9/8)$. Therefore, we can also write $\mathcal{O}\left(2^{\text{poly}(\log(n))}\right) \subset \mathcal{O}\left((9/8)^{\frac{n}{m}}\right)$. In addition, if we have $c_1^2 R \in \mathcal{O}(2^n)$, from (120), one can obtain

$$F_n(L,l) \in \frac{1}{3^{\frac{n}{m}} 2^{\left(2-\frac{3}{m}\right)n}} \mathcal{O}\left((9/8)^{\frac{n}{m}}\right) \mathcal{O}(2^n) = \mathcal{O}\left(\frac{1}{2^{\left(1-\frac{1}{m}\log_2 3\right)n}}\right)\,, \tag{138}$$

where $1 - \frac{1}{m}\log_2 3 > 0$ for $m \geqslant 2$. Hence the upper bound of $\text{Var}[\partial_\nu C_G]$ exponentially vanishing when $m \geqslant 2$. □

Let us now consider case (ii) in Theorem 1.

Proof. We now have $2^{2m(L-l+1)+1} \in \mathcal{O}\left(2^{\text{poly}(\log(n))}\right) \subset \mathcal{O}\left((9/8)^{\frac{n}{m}}\right)$. We have from Eq. (135) that if $c_i, c_j \in \mathcal{O}(1)$, the following bound holds

$$F_n(L,l) \in \frac{1}{3^{\frac{2n}{m}} 2^{\left(3-\frac{4}{m}\right)n}} \mathcal{O}\left((9/8)^{\frac{n}{m}}\right) \mathcal{O}\left(2^{2n}\right) = \mathcal{O}\left(\frac{1}{2^{\left(1-\frac{1}{m}\right)n}}\right).$$

Hence, the upper bound of $\text{Var}[\partial_\nu C_G]$ exponentially vanishing when $m \geqslant 2$. □

B. Proof of Corollary 2

Corollary 2. Consider the function $F_n(L,l)$. Let O be an operator of the form (68), as in Theorem 2. If at least one term $c_i^2 \epsilon(\rho_{k,k'})\epsilon(\widehat{O}_i)$ in the sum in (70) vanishes no faster than $\Omega(1/\operatorname{poly}(n))$, and if the number of layers L is $\mathcal{O}(\log(n))$, then

$$G_n(L,l) \in \Omega\left(\frac{1}{\text{poly}(n)}\right). \tag{139}$$

On the other hand, if at least one term $c_i^2 \epsilon(\rho_{k,k'})\epsilon(\widehat{O}_i)$ in the sum in (70) vanishes no faster than $\Omega\left(1/2^{\text{poly}(\log(n))}\right)$, and if the number of layers is $\mathcal{O}(\text{poly}(\log(n)))$, then

$$G_n(L,l) \in \Omega\left(\frac{1}{2^{\text{poly}(\log(n))}}\right). \tag{140}$$

Proof. Let us assume that at least one term $c_i^2 \epsilon(\rho_{k,k'})\epsilon(\widehat{O}_i)$ in (70) vanishes no faster than $\Omega(1/\operatorname{poly}(n))$. Then, if we also assume $L \in \mathcal{O}(\log(n))$, we have $(2^m+1)^{-(L+l)} \in \Omega(1/\operatorname{poly}(n))$. The latter implies

$$G_n(L,l) \in \Omega\left(\frac{1}{\text{poly}(n)}\right)\Omega\left(\frac{1}{\text{poly}(n)}\right) = \Omega\left(\frac{1}{\text{poly}(n)}\right).$$

On the other hand, if at least one term $c_i^2 \epsilon(\rho_{k,k'})\epsilon(\widehat{O}_i)$ in (70) vanishes no faster than $\Omega\left(1/2^{\text{poly}(\log(n))}\right)$, and if $L \in \mathcal{O}(\text{poly}(\log(n)))$, we have $(2^m+1)^{-(L+l)} \in \Omega\left(1/2^{\text{poly}(\log(n))}\right)$. Therefore, we obtain

$$G_n(L,l) \in \Omega\left(\frac{1}{2^{\text{poly}(\log(n))}}\right)\Omega\left(\frac{1}{2^{\text{poly}(\log(n))}}\right) = \Omega\left(\frac{1}{2^{\text{poly}(\log(n))}}\right).$$

□

Supplementary Note 9: Faithfulness of local cost function for Quantum autoencoder

Recall that the global cost function (C'_G) and local cost function (C'_L) for the quantum autoencoder are defined as

$$C'_G = 1 - \text{Tr}\left[|\mathbf{0}\rangle\langle\mathbf{0}|\rho_\text{B}^\text{out}\right]$$
$$C'_\text{L} = 1 - \frac{1}{n_\text{B}}\sum_{j=1}^{n_\text{B}} \text{Tr}\left[\left(|0\rangle\langle 0|_j \otimes \mathbb{1}_{\bar{j}}\right)\rho_\text{B}^\text{out}\right].$$

We relate the C'_L and C'_G cost functions in the following proposition. Because this proposition establishes that C'_L and C'_G vanish under the same conditions, and because C'_G is faithful [7], this in turn proves that C'_L is a faithful cost function.

Proposition 3. The cost functions for the quantum autoencoder satisfy

$$C'_\text{L} \leqslant C'_G \leqslant n_\text{B} C'_\text{L}. \tag{141}$$

Proof. Let us first prove $C'_{\rm L} \leqslant C'_G$. Given the state $\rho_{\rm B}^{\rm out}$, we can define E_j as the event of qubit j being measured on the $|0\rangle_j$ state, such that the probability of E_j is given by $\Pr(E_j) = {\rm Tr}\left[\widehat{O}_j^L \rho_{\rm B}^{\rm out}\right]$, where $\widehat{O}_j^L = |0\rangle\langle 0|_j \otimes \mathbb{1}_{\bar{j}}$. Then, we can write

$$C'_G = 1 - \Pr\left(\bigcap_{j=1}^{n_{\rm B}} E_j\right) \tag{142}$$

Similarly, for the local cost function, we have

$$C'_{\rm L} = 1 - \frac{1}{n_{\rm B}} \sum_{j=1}^{n_{\rm B}} \Pr(E_j)\,. \tag{143}$$

Then, it is known that for any set of events $\{E_1, \cdots, E_{n_{\rm B}}\}$, the following property always holds

$$1 - \Pr\left(\bigcap_{j=1}^{n_{\rm B}} E_j\right) \geq \frac{1}{n_{\rm B}} \sum_{j=1}^{n_{\rm B}} \left(1 - \Pr(E_j)\right)\,. \tag{144}$$

From the definition of C'_G and $C'_{\rm L}$ in Eqs. (142)– (143), and from Eq. (144) we have

$$C'_{\rm L} \leqslant C'_G\,. \tag{145}$$

Next, let us prove $C'_G \leqslant n_{\rm B} C'_{\rm L}$. Since for any set of events $\{E_1, \cdots, E_{n_{\rm B}}\}$, we have

$$1 - \Pr\left(\bigcap_{j=1}^{n_{\rm B}} E_j\right) \leqslant \sum_{j=1}^{n_{\rm B}} \left(1 - \Pr(E_j)\right). \tag{146}$$

By definition, we finally have

$$C'_G \leqslant n_{\rm B} C'_{\rm L}. \tag{147}$$

Combining Eqs. (145) and (147), we get (141), which indicates $C'_{\rm L} = 0 \iff C'_G = 0$. □

[1] Benoît Collins and Piotr Śniady, "Integration with respect to the haar measure on unitary, orthogonal and symplectic group," Communications in Mathematical Physics **264**, 773–795 (2006).
[2] Zbigniew Puchała and Jaroslaw Adam Miszczak, "Symbolic integration with respect to the haar measure on the unitary groups," Bulletin of the Polish Academy of Sciences Technical Sciences **65**, 21–27 (2017).
[3] Christoph Dankert, Richard Cleve, Joseph Emerson, and Etera Livine, "Exact and approximate unitary 2-designs and their application to fidelity estimation," Physical Review A **80**, 012304 (2009).
[4] R. E. A. C. Paley and A. Zygmund, "A note on analytic functions in the unit circle," Math. Proc. Camb. Phil. Soc. **28**, 266 (1932).
[5] Motohisa Fukuda, Robert König, and Ion Nechita, "RTNI—a symbolic integrator for haar-random tensor networks," Journal of Physics A: Mathematical and Theoretical **52**, 425303 (2019).
[6] M. A. Nielsen and I. L. Chuang, *Quantum Computation and Quantum Information: 10th Anniversary Edition*, 10th ed. (Cambridge University Press, New York, NY, USA, 2011).
[7] J. Romero, J. P. Olson, and A. Aspuru-Guzik, "Quantum autoencoders for efficient compression of quantum data," Quantum Science and Technology **2**, 045001 (2017).
